# Nanoparticles Synergize Ferroptosis and Cuproptosis to Potentiate Cancer Immunotherapy

**DOI:** 10.1002/advs.202310309

**Published:** 2024-03-13

**Authors:** Youyou Li, Jing Liu, Yimei Chen, Ralph R. Weichselbaum, Wenbin Lin

**Affiliations:** ^1^ Department of Chemistry The University of Chicago Chicago IL 60637 USA; ^2^ Department of Radiation and Cellular Oncology and Ludwig Center for Metastasis Research The University of Chicago Chicago IL 60637 USA

**Keywords:** cuproptosis, ferroptosis, immunotherapy, nanoparticles

## Abstract

The recent discovery of copper‐mediated and mitochondrion‐dependent cuproptosis has aroused strong interest in harnessing this novel mechanism of cell death for cancer therapy. Here the design of a core‐shell nanoparticle, CuP/Er, for the co‐delivery of copper (Cu) and erastin (Er) to cancer cells for synergistic cuproptosis and ferroptosis is reported. The anti‐Warburg effect of Er sensitizes tumor cells to Cu‐mediated cuproptosis, leading to irreparable mitochondrial damage by depleting glutathione and enhancing lipid peroxidation. CuP/Er induces strong immunogenic cell death, enhances antigen presentation, and upregulates programmed death‐ligand 1 expression. Consequently, CuP/Er promotes proliferation and infiltration of T cells, and when combined with immune checkpoint blockade, effectively reinvigorates T cells to mediate the regression of murine colon adenocarcinoma and triple‐negative breast cancer and prevent tumor metastasis. This study suggests a unique opportunity to synergize cuproptosis and ferroptosis with combination therapy nanoparticles to elicit strong antitumor effects and potentiate current cancer immunotherapies.

## Graphical abstract

This paper reports a core‐shell nanoparticle, CuP/Er, for the co‐delivery of copper (Cu) and erastin (Er) to cancer cells for synergistic cuproptosis and ferroptosis. CuP/Er induces strong immunogenic cell death, and when combined with immune checkpoint blockade, effectively reinvigorates T cells to mediate the regression of murine colon adenocarcinoma and triple‐negative breast cancer and prevent tumor metastasis.

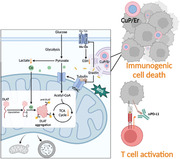

## 1 Introduction

Cancer cells resistant to apoptosis and necroptosis are difficult to kill by chemotherapy and radiotherapy.^[^ ^1^ ^]^ Discovery of new cell death pathways is crucial for eliminating these resistant cells.^[^ ^2^ ^]^ In 2022, Tsvetkov et al. discovered a new form of regulated cell death that is termed cuproptosis.^[^ ^3^ ^]^ Unlike traditional programmed cell death pathways, cuproptosis is copper (Cu)‐ and mitochondrial respiration‐dependent.^[^ ^3^ ^]^ By directly binding Cu to lipoylated components of the tricarboxylic acid (TCA) cycle, cuproptosis causes aggregation of lipoylated proteins and loss of iron‐sulfur cluster proteins to create proteotoxic stress and elicit cell death. However, it is challenging to exploit cuproptosis for cancer therapy due to its dependence on the TCA cycle.^[^ ^4^ ^]^ Cancer cells also exhibit the hallmark Warburg effect with higher glucose uptake and by fermenting glucose to lactate as fuels.^[^ ^5^, ^6^ ^]^ As lactate ^[^ ^7^ ^]^ and glutamine ^[^ ^8^ ^]^ provide major fuels for the TCA cycle, the TCA cycle plays a complex role in cancer metabolism, redox balance, and tumorigenesis.^[^ ^9^, ^10^, ^11^, ^12^ ^]^ Thus, cancer treatments targeting both aerobic glycolysis and mitochondrial metabolism will likely be more effective. Treatments that sensitize cancer cells to cuproptosis can leverage this novel form of cancer death for effective cancer therapy.

Erastin (Er) was found to selectively kill cancer cells overexpressing Small T and RAS oncoproteins in 2003.^[^ ^13^ ^]^ Er was shown by Dixon et al. in 2012 to induce ferroptosis ^[^ ^14^ ^]^ by depleting glutathione (GSH) via inhibiting the antiporter system X_c_^−^. ^[^ ^15^ ^]^ Er also opens voltage‐dependent anion channels (VDACs) on mitochondrial outer membranes,^[^ ^16^ ^]^ leading to mitochondrial membrane potential (Δψ) change, decrease in glycolysis, and increase of ROS production. Er has been chemically modified to increase aqueous solubility and bioavailability,^[^ ^17^, ^18^ ^]^ but as a monotherapy, Er and its analogs do not efficiently inhibit tumor growth in vivo. We surmised that Er could be combined with other therapeutics to potentiate its antitumor efficacy without increasing general toxicity. Because of some nanoparticles’ ability to accumulate in tumors via the enhanced permeability and retention (EPR) effect,^[^ ^19^, ^20^, ^21^, ^22^, ^23^, ^24^, ^25^, ^26^, ^27^ ^]^ we were intrigued by the possibility of combining Cu‐mediated cuprotosis and Er‐mediated ferroptosis in a nanoparticle to improve tumor targeting and enhance antitumor efficacy.

As a new class of hybrid core‐shell nanoparticles, nanoscale coordination polymers (NCPs) can incorporate hydrophilic molecules in the core and hydrophobic molecules on the shell to significantly improve their blood circulation and tumor accumulation, providing a versatile platform for delivering synergistic combination treatments.^[^ ^28^, ^29^, ^30^, ^31^ ^]^ Since nanoparticle‐mediated cuproptosis was recently shown to up‐regulate programmed death‐ligand 1 (PD‐L1) expression of cancer cells and reprogram the immunosuppressive tumor microenvironment (TME) ^[^ ^32^, ^33^, ^34^ ^]^ and cuproptosis can be enhanced by ferroptosis inducers,^[^ ^35^ ^]^ we anticipate that NCP‐mediated ferroptosis and cuproptosis will synergize with immune checkpoint blockade (ICB) to elicit potent antitumor immunity.

Herein, we report the development of a novel bifunctional CuP/Er NCP nanoparticle, comprising copper ions and peroxide in the core and Er on the shell, to harness the synergistic effects of cuproptosis and ferroptosis. CuP/Er exerts dual effects of sensitizing tumor cells to cuproptosis by reducing their reliance on aerobic glycolysis and inhibiting the TCA cycle through the induction of oligomerization in lipoylated TCA proteins in mitochondria and to ferroptosis by increasing ROS production and intracellular redox imbalance. As a result, CuP/Er depleted glutathione (GSH), enhanced lipid peroxidation, and caused irreparable mitochondrial damages, leading to robust inhibition of tumor growth in mouse models of breast and colon cancer. CuP/Er treatment induced potent immunogenetic cell death to enhance antigen presentation and upregulated PD‐L1 expression in tumor cells. The combination of CuP/Er with an anti‐PD‐L1 antibody (αPD‐L1) potently regressed tumors and prevented tumor metastasis via synergizing T cell proliferation by CuP/Er and T cell reinvigoration by ICB to afford potent cancer immunotherapy.

## 2 Results

### 2.1 Synthesis and Characterization of CuP/Er

**Figure 1:**
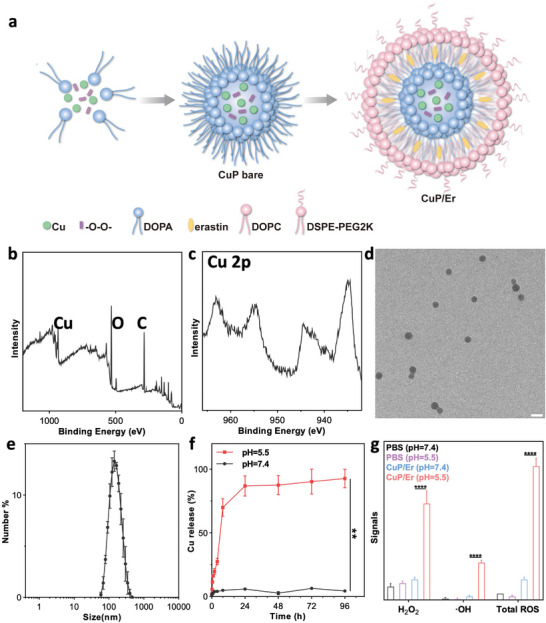
Synthesis and characterization of CuP/Er. a) Schematic showing the synthesis of CuP‐bare and CuP/Er. CuP/Er was synthesized by coating CuP‐bare particles with a lipid bilayer and Er. b) Survey XPS spectrum and c) Cu 2p XPS spectrum of CuP‐bare particles. d) TEM image of CuP/Er. Scale bar is 200 nm. e) Size distribution of CuP/Er by DLS. f) Cu release from CuP/Er in PBS at pH 5.5 and 7.4. g) ROS generation by CuP/Er in PBS at pH 5.5 and 7.4. Data are represented as mean ± SD, n = 3 in e), f), and g).

The NCP particle encapsulating cupric ions and peroxide groups, CuP‐bare, was synthesized by mixing a reverse microemulsion of Na_3_PO_4_ and 1,2‐dioleoyl‐*sn*‐glycero‐3‐phosphate (DOPA) and a reverse microemulsion CuCl_2_ and H_2_O_2_ under vigorous stirring (**Figure** **1**a; Figure S1, Supporting Information).^[^ ^31^ ^]^ We used X‐ray photoelectron spectroscopy (XPS), gas chromatography (GC), and inductively coupled plasma‐mass spectrometry (ICP‐MS) to characterize CuP‐bare. The Cu 2p XPS spectrum of CuP‐bare showed two main peaks at 935.2 and 955.3 eV and two satellite peaks at 942.9 and 962.6 eV (Figure 1b,c), consistent with the presence of Cu(II) ions. The O 1s XPS spectrum of CuP‐bare was fitted into two main peaks at 531.4 and 532.9 eV (Figure S2, Supporting Information), which were assigned to oxygen atoms from phosphate and peroxide (‐O─O‐) groups, respectively.^[^ ^36^ ^]^ The presence of peroxide was supported by GC quantification of oxygen gas generated from oxidation of CuP (see below) by KMnO_4_ (Figure S3 and Table S1, Supporting Information). CuP showed a copper to peroxide ratio of 1.50 ± 0.02 based on ICP‐MS analysis of Cu and GC quantification of peroxide groups. Based on these results, we proposed a molecular formula of Cu_3_(O─O)_2_(HPO_4_) for the coordination polymer core of CuP‐bare. CuP‐bare had a Z‐average diameter of 78.4 ± 0.6 nm and a polydispersity index (PDI) of 0.19 ± 0.01 by dynamic light scattering (DLS).

CuP‐bare particles were coated with 1,2‐dioleoyl‐sn‐glycero‐3‐phosphocholine (DOPC), cholesterol, and DSPE‐PEG2K to obtain the core‐shell particle CuP. By adding one equivalent of Er relative to Cu in the coating process, we obtained the bifunctional particle CuP/Er. CuP/Er had a Z‐average diameter of 146.8 ± 1.6 nm and a PDI of 0.24 ± 0.03 by DLS (Figure 1e). Transmission electron microscopy (TEM) imaging showed a spherical morphology for CuP/Er (Figure 1d). CuP/Er did not release Cu at pH 7.4 but quickly released Cu at pH 5.5 (Figure 1f), indicating the ability of CuP/Er to trigger release Cu(II) ions in acidic endo/lysosome environments. CuP/Er showed pH‐dependent ROS generation, with a 3.7‐fold higher H_2_O_2_ signal and a 9.4‐fold higher hydroxy radical (^.^OH) signal at pH 5.5 over pH 7.4 (Figure 1g). This result suggests the potential of triggering cancer cell death via ROS generation in the acidic TME.

### 2.2 Release of Cu and Er Inside Cancer Cells

**Figure 2:**
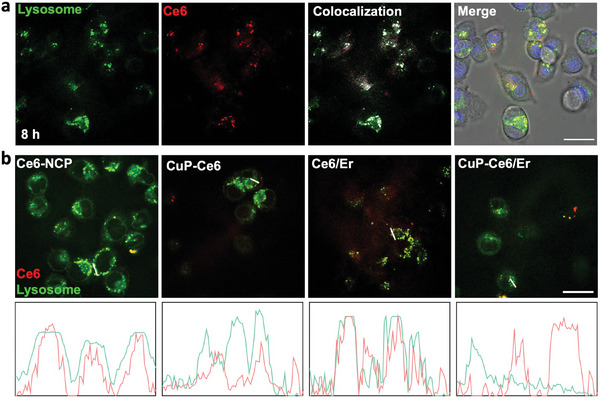
Cellular colocalization and endo/lysosomal escape of CuP/Er. a) LysoTracker and Ce6 colocalization after incubation of 4T1 cells with Ce6‐NCP for 8 h. b) LysoTracker and Ce6 colocalization after incubation of 4T1 cells with Ce6‐NCP, CuP‐Ce6, Ce6/Er, and CuP‐Ce6/Er for 8 h. Line profiles of Ce6 and LysoTracker along the white lines showed the detailed overlays of LysoTracker and Ce6. Scale bars are 20 µm.

Triple‐negative breast cancer 4T1 cells showed time‐dependent uptake of CuP/Er over 24 h (Figure S4, Supporting Information). We synthesized an NCP particle with only Ce6 in the core (Ce6‐NCP) and Er‐NCP, CuP, and CuP/Er particles with Ce6 incorporated in the cores (Ce6/Er, CuP‐Ce6 and CuP‐Ce6/Er, respectively) to study the cellular uptake process. After incubation for different lengths of time, 4T1 cells were stained with LysoTracker. Ce6‐NCP colocalized with LysoTracker with colocalization coefficients of 0.73 and 0.96 at 3 and 8 h, respectively (**Figure** **2**a; Figure S5, Supporting Information). This result suggests the endocytosis of Ce6‐NCP particles and their trapping in the endo/lysosomes. In contrast, CuP‐Ce6 showed significantly reduced colocalization with Lysotracker while Ce6/Er showed slightly reduced colocalization with Lysotracker (Figure 2b), indicating the disruption of endo/lysosome membranes and the distribution of NCP particles into the cytoplasm. Importantly, CuP‐Ce6/Er did not show any colocalization with Lysotracker. This result suggests a strong synergy between CuP and Er in ROS generation and endo/lysosome membrane disruption, leading to efficient endo/lysosomal escape of CuP‐Ce6/Er particles. We also observed gradual Cu accumulation in the mitochondria of 4T1 cells treated with CuP/Er (Figure S6, Supporting Information).

### 2.3 CuP/Er Enhances Ferroptosis via ROS Generation, Lipid Peroxidation, and GSH Depletion

**Figure 3:**
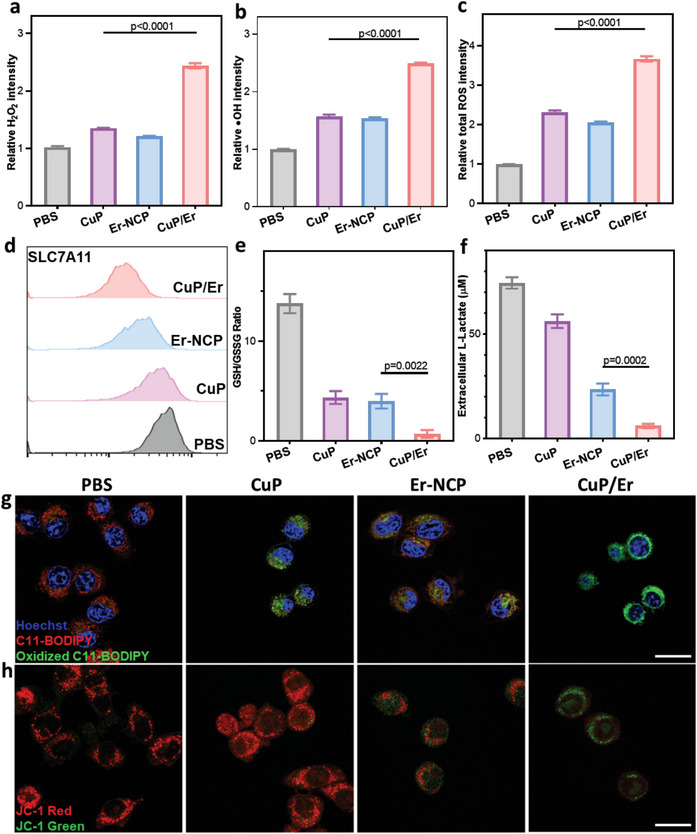
CuP/Er induces ferroptosis and reduces glycolysis. a) H_2_O_2_, ^.^b) OH, and c) total ROS levels in 4T1 cells after different treatments. d) Representative flow cytometric plots of SLC7A11 in 4T1 cells after different treatments. e) GSH/GSSG ratios in 4T1 cells after different treatments. f) Extracellular *L*‐lactate levels of 4T1 cells after different treatments. g) Lipid peroxidation of 4T1 cells after different treatments by C11‐BODIPY‐581/591 staining. h) Mitochondrial membrane potential change by JC‐1 staining in 4T1 cells after different treatments. Scale bars are 20 µm in g) and h). All data are represented as mean ± SD, n = 3.

We used flow cytometry to determine the ROS levels in 4T1 cells. H_2_O_2_, hydroxyl radical (^•^OH), and total ROS were detected by hydrogen peroxide assay kit, aminophenyl fluorescein (APF) kit, and dichlorodihydrofluorescein diacetate (H_2_DCFDA) kit, respectively. CuP/Er treatment increased H_2_O_2_, ^•^OH, and total ROS levels by 2.4‐, 2.5‐, and 3.7‐fold, respectively, over PBS control. In comparison, CuP treatment increased H_2_O_2_, ^•^OH, and total ROS levels by 1.4‐, 1.6‐, and 2.1‐fold, respectively, whereas Er‐NCP treatment increased H_2_O_2_, ^•^OH, and total ROS levels by 1.2‐, 1.5‐, and 2.3‐fold, respectively (**Figure** **3**a–c). These results show that Cu and Er from CuP/Er synergistically enhance ROS generation.

We next determined lipid peroxidation in 4T1 cells using C11‐BODIPY‐581/591 dye. The oxidized C11‐BODIPY‐581/591 shifts its fluorescence from red to green, and the percentage of green signals represents the percentage of oxidized C11‐BODIPY. CuP/Er treatment gave 87.1% oxidized C11‐BODIPY, while CuP and Er‐NCP treatments showed 49.9% and 33.8% oxidized C11‐BODIPY, respectively (Figure 3g; Figure S7b, Supporting Information). These results indicate that CuP/Er enhances lipid peroxidation in 4T1 cells. We also observed reduced GPX4 expression in CuP/Er treated cells (Figure S8, Supporting Information). We also performed ROS generation, lipid peroxidation, and cytotoxicity assays in the presence of ferrostatin 1 (Fer‐1), UK5099, or Z‐VAD‐FMK. The results showed that both ferroptosis inhibitors and cuproptosis inhibitors reduced ROS generation, lipid peroxidation, and cytotoxic effects of CuP/Er, while the apoptosis inhibitor had no effect on the ROS generation, lipid peroxidation, and cytotoxicity of CuP/Er (Figure S14, Supporting Information).

Er is known to reduce SLC7A11 expression and cause ferroptosis.^[^ ^17^ ^]^ CuP/Er treatment reduced the expression of SLC7A11 in 4T1 cells to 30.4% of PBS control. In comparison, CuP and Er‐NCP treatments reduced SLC7A11 expression to 79.6% and 53.0% of PBS control, respectively (Figure 3d; Figure S7a, Supporting Information). CuP, Er‐NCP, and CuP/Er treatments greatly decreased the GSH/glutathione disulfide (GSSG) ratio from 13.8 for PBS control to 4.4, 4.0, and 0.7, respectively (Figure 3e). These results show that both CuP and Er‐NCP caused redox imbalance in 4T1 cells via cuproptosis and ferroptosis, respectively, and CuP/Er further exacerbates redox imbalance via synergistic cuproptosis and ferroptosis.^[^ ^3^, ^17^ ^]^

### 2.4 CuP/Er Disrupts Mitochondrial Membranes and Decreases Glycolysis

Er is known to bind to VDAC proteins to induce mitochondrial dysfunction as manifested by Δψ changes.^[^ ^16^ ^]^ JC‐1 is a sensitive marker for probing Δψ loss by displaying green signals from the JC‐1 monomer. CuP, Er‐NCP, and CuP/Er treatments of 4T1 cells increased the percentages of green signals from 15.7% for PBS control to 21.9%, 46.4%, and 67.1%, respectively (Figure 3h; Figure S7c, Supporting Information). This result indicates that CuP/Er synergistically disrupts mitochondrial function.

Erastin was also reported to decrease glycolysis and reverse the Warburg effect of cancer cells.^[^ ^37^ ^]^ We determined intracellular and extracellular *L*‐lactate levels in 4T1 cells by *L*‐lactate assay. CuP, Er‐NCP, and CuP/Er treatments slightly reduced intracellular *L*‐lactate levels by 3.5%, 10.9%, and 24.4%, respectively, but greatly reduced extracellular *L*‐lactate levels by 24.6%, 68.4%, and 92.1%, respectively (Figure 3f; Figure S9, Supporting Information). These results indicate the strong anti‐Warburg effect of CuP/Er on cancer cells.

### 2.5 CuP/Er Induces Cuproptosis and Exhausts Mitochondrial Metabolism

**Figure 4:**
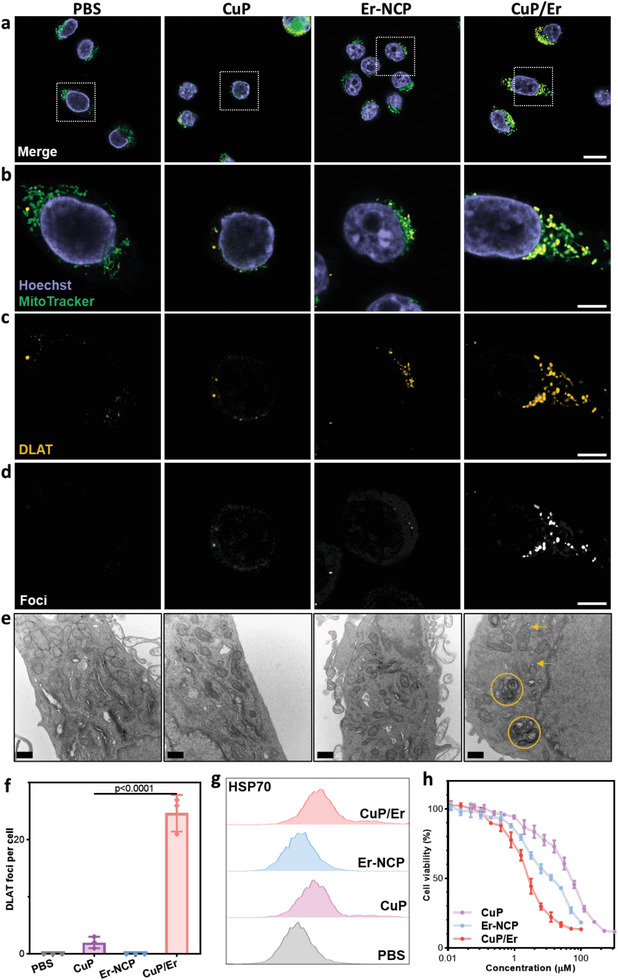
CuP/Er induces cuproptosis and exhausts mitochondrial metabolism. a) Merged images of DLAT and MitoTracker staining of 4T1 cells after different treatments. b) Zoomed‐in images of the white frames in a) and DLAT channel c). d) Foci masks of DLAT channels. Scale bars are 20 µm in a) and 5 µm in b), c) and d). e) TEM images of 4T1 cells after different treatments. Circles represent mitophagy and arrows represent mitochondria‐derived vesicles. Scale bars are 200 nm. f) DLAT foci number per tumor cell by analyzing DLAT staining in confocal images in a). g) Representative flow cytometric plots of HSP70 in 4T1 cells after different treatments. h) 4T1 cell viability after different treatments. All data are represented as mean ± SD, n = 3.

Cuproptosis is mediated by FDX1 and lipoylation of TCA cycle proteins.^[^ ^3^ ^]^ FDX1 expression in cells treated with CuP/Er was significantly decreasing (Figure S8, Supporting Information). Lipoylation of dihydrolipoamide acetyltransferase (DLAT) can be observed by immunofluorescence. CuP/Er treatment significantly induced DLAT foci in 4T1 cells (**Figure** **4**a–d; Figure S10 and S11, Movie S1 for DLAT signals and Movie S2, Supporting Information for DLAT Foci for CuP/Er‐treated cells) and markedly increased DLAT foci number per cell (Figure 4f). This result indicates efficient induction of cuproptosis by CuP/Er.

HSP70 protein is an indicator for acute proteotoxic stress and greatly increases in cancer cells undergoing cuproptosis.^[^ ^3^ ^]^ CuP/Er and CuP treatments increased HSP70 expression in 4T1 cells by 2.22‐ and 1.71‐fold, respectively, over PBS control (Figure 4g; Figure S7d, Supporting Information). TEM imaging showed that only CuP/Er‐treated 4T1 cells displayed mitochondrion‐derived vesicles and mitophagy (Figure 4e), indicating extremely abnormal mitochondrial metabolism after CuP/Er treatment. Consequently, CuP/Er exhibited potent cytotoxicity against 4T1 cells with a half‐maximal inhibitory concentration (IC_50_) of 2.9 ± 0.3 µM, compared to IC_50_ values of 45.7 ± 6.6 µM and 11.4 ± 2.2 µM for CuP and Er‐NCP, respectively (Figure 4h). CuP/Er showed a combination index of 0.32, indicating a strong synergy between Er‐induced ferroptosis and CuP‐mediated cuproptosis. While the empty NCP did not show apparent cytotoxicity on 4T1 cells and HEK293T cells (Figures S12 and S13, Supporting Information), other particles showed comparable cytotoxicity on HEK293T cells as on 4T1 cells. Cell death induced by CuP/Er was partly rescued by ferroptosis inhibitor and cuproptosis inhibitor, while apoptosis inhibitor did not improve cell viability (Figure S14, Supporting Information).

### 2.6 CuP/Er Induces ICD, Promotes Antigen Presentation, and Upregulates PD‐L1 Expression

**Figure 5:**
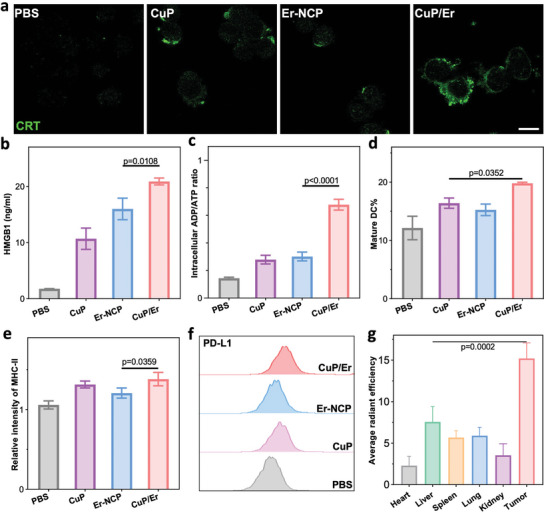
CuP/Er induces ICD, promotes antigen presentation, and upregulates PD‐L1 expression. a) Confocal images of CRT expression in 4T1 cells after different treatments. Scale bar is 20 µm. b) HMGB1 concentrations in 4T1 cell media after different treatments. c) Intracellular ADP/ATP ratios in 4T1 cells after different treatments. d) Mature DC percentages and e) relative MHC II intensity in BMDCs co‐incubated with pre‐treated 4T1 cells. f) Representative flow cytometric plots of PD‐L1 expression in 4T1 cells after different treatments. g) Average Ce6 radiant efficiency of different organs 24 h after i.v. injection of CuP‐Ce6/Er to 4T1 tumor‐bearing mice. All data are represented as mean ± SD, n = 3. The unit in g) is ×10^8^ [p s^−1^ cm^−2^ per sr]/[µW cm^−2^].

ICD is characterized by release of calreticulin (CRT), high mobility group box 1 (HMGB1), and adenosine 5′‐triphosphate (ATP) as major damaged associated molecular patterns (DAMPs).^[^ ^38^ ^]^ CuP/Er induced higher CRT expression and increased HMGB1 release and intracellular adenosine diphosphate (ADP)/ATP ratio by 11.7‐ and 4.7‐fold, respectively, over PBS control in 4T1 cells (**Figure** **5**a–c; Figure S7e, Supporting Information). The addition of a ferroptosis or cuproptosis inhibitor greatly suppressed CRT expression, HMGB1 release, and intracellular ADP/ATP ratio induced by CuP/Er treatment (Figure S16, Supporting Information).

DAMPs released from tumor cells are sensed by antigen presenting cells (APCs) to present antigens to T cells to stimulate adaptive immunity.^[^ ^39^ ^]^ APCs expressing major histocompatibility complex (MHC) proteins can stimulate CD4^+^ helper T cells as well as cytotoxic CD8^+^ T cells.^[^ ^40^, ^41^ ^]^ Bone marrow derived dendritic cells (BMDCs) were co‐incubated with 4T1 cells pre‐treated with CuP, Er‐NCP, or CuP/Er to evaluate DC maturation and the expression of MHC II proteins induced by the DAMPs from treated 4T1 cells. Mature DCs (CD80^+^CD86^+^) increased from 12.3% in PBS control to 19.8% in the CuP/Er group (Figure 5d). CuP/Er treatment increased the expression of MHC II proteins by 1.4‐fold over PBS control (Figure 5e). These results show that the released DAMPs from CuP/Er‐treated 4T1 cells facilitate DC maturation and antigen presentation.

The effect of CuP/Er on PD‐L1 expression in 4T1 cells in vitro was evaluated by flow cytometry. CuP, Er‐NCP, and CuP/Er significantly increased PD‐L1 expression in 4T1 cells by 1.4‐, 1.1‐ and 1.5‐fold, respectively, over PBS control (Figure 5f; Figure S7f, Supporting Information). Increased PD‐L1 expression by CuP, Er‐NCP, and CuP/Er was supported by immunofluorescence of tumor slides (Figure S22, Supporting Information). The increased PD‐L1 expression in 4T1 cells can inhibit T cell responses and facilitate immune evasion of tumor cells,^[^ ^42^ ^]^ which suggests that CuP/Er can be combined with ICB to reinvigorate T cell‐mediated immune response for effective cancer immunotherapy.

### 2.7 Tumor Targeting by CuP/Er

To evaluate the ability of CuP/Er to target tumors, we determined biodistribution of intravenously (i.v.) injected CuP‐Ce6/Er into subcutaneous 4T1 tumor‐bearing mice. Ce6 signals gradually increased in the tumors over 24 h (Figures S17 and S18, Supporting Information). The mice were sacrificed at 24 h post‐injection, and the tumors and main organs were excised for *ex vivo* imaging. The tumors had the highest signals compared to other organs, with 6.6‐, 4.3‐, 2.7‐, 2.6‐, and 2.0‐fold higher Ce6 signals than the hearts, kidneys, spleens, lungs, and livers, respectively (Figure 5g; Figure S19, Supporting Information). The circulating half‐lives of Ce6 and Cu in plasma were determined as 8.0 ± 2.5 and 3.6 ± 0.6 h, respectively, by IVIS and ICP‐MS (Figures S20 and S21, Tables S2 and S3, Supporting Information). These results indicate the tumor targeting ability of CuP/Er for efficient co‐delivery of Er and Cu ions to the tumors in vivo.

### 2.8 Anticancer Efficacy and Immune Responses of CuP/Er Plus Immune Checkpoint Blockade

**Figure 6:**
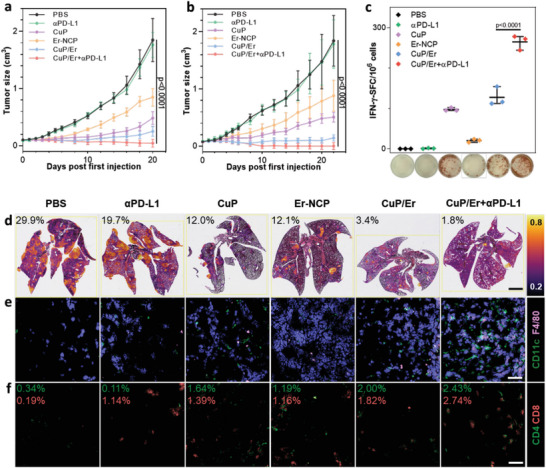
In vivo anticancer efficacy and immune responses of CuP/Er plus αPD‐L1. a) Growth curves of MC38 tumors after different treatments. Data are represented as mean ± SD, n = 6. b) Growth curves of 4T1 tumors after different treatments. Data are represented as mean ± SD, n = 3 in PBS and αPD‐L1 groups and n = 6 in other groups. c) ELISpot assay of IFN‐γ‐SFCs 10^−6^ splenocytes and representative images of formed spots in MC38 tumor‐bearing mice after different treatments. n = 3. d) Colormaps of nucleus/cell ratios with H&E staining of lungs in 4T1 tumor‐bearing mice after different treatments. The color bar represents the nucleus/cell ratio from 0.2 to 0.8. e) CD11c and F4/80 and f) CD4 and CD8 immunofluorescence staining of cryo‐sectioned 4T1 tumor slides after different treatments. Scale bar is 2 mm in d) and 40 µm in e) and f).

The in vivo antitumor activity was first evaluated on the syngeneic MC38 colon cancer model. Seven days after subcutaneous inoculation of MC38 cells, C57BL/6 mice with ≈100 mm^3^ tumors were randomized and i.v. injected with PBS, CuP, Er‐NCP, or CuP/Er at a dose of 3.5 mg kg^−1^ Cu or/and 30 mg kg^−1^ Er once every three days for a total of three doses. For αPD‐L1 groups, the mice were intraperitoneally injected with 75 ug per mice αPD‐L1 immediately after i.v. treatments. While CuP and Er‐NCP slowed tumor growth with tumor growth inhibition indices (TGIs) of 73.9% and 54.2%, respectively, CuP/Er inhibited tumor growth with a TGI of 86.5% (**Figure** **6**a). Interestingly, although the low dose of αPD‐L1 was ineffective with a TGI of 4.5%, the combination of CuP/Er and αPD‐L1 effectively regressed the tumors with a TGI of 97.7%. These results indicate the synergistic effects of Er and Cu ions delivered by CuP/Er and suggest reversal of immunosuppression by αPD‐L1 to stimulate the host immune response and enhance the antitumor efficacy of CuP/Er. The mice in different groups did not show obvious body weight losses throughout the treatment (Figure S28, Supporting Information).

**Figure 7:**
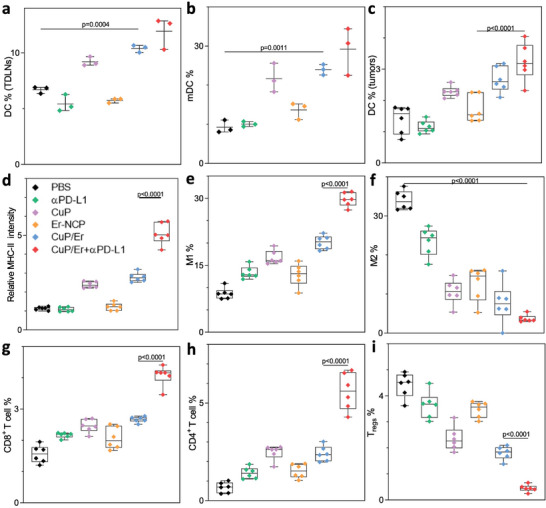
Quantification of immune cells in MC38 tumor‐bearing mice. a) percentages of DCs (gated on CD11b^+^CD11c^+^) of total cells and b) percentages of mDCs (gated on CD11b^+^CD11c^+^CD80^+^CD86^+^) of total DCs in the TDLNs; c) Percentages of DCs (gated on CD11b^+^CD11c^+^) of total cells in the tumors; d) Relative MHC II intensity of DCs in the tumors; Percentages of e) M1 (gated on CD11b^+^F4/80^+^CD86^+^CD206^−^) and f) M2 (gated on CD11b^+^F4/80^+^CD206^+^CD86^−^) macrophages of total macrophages in the tumors; Percentages of g) Cytotoxic T cells (gated on CD3ε^+^CD8^+^), helper T cells (gated on CD3ε^+^CD4^+^) and T_regs_ (gated on CD3ε^+^CD4^+^Foxp3^+^) of total cells in the tumors; Data: n = 3 in a) and b), n = 6 in c‐i).

We used flow cytometry to investigate the antitumor immune response by profiling immune cells in the tumors and tumor‐draining lymph nodes (TDLNs) in MC38 tumor‐bearing mice 2 days post the last treatment. Er‐NCP and αPD‐L1 treatments did not significantly impact the percentages of DCs and mature DCs in the TDLNs, while CuP, CuP/Er, and CuP/Er plus αPD‐L1 treatments increased DC percentages from 6.7% for PBS to 9.2%, 10.4%, and 11.9%, respectively and mature DC percentages from 9.2% for PBS to 21.7%, 24.0%, and 29.2%, respectively (**Figure** **7**a,b). Similarly, Er‐NCP and αPD‐L1 treatments did not significantly impact the percentages of DCs and MHC II expression in MC38 tumors, but CuP, CuP/Er, and CuP/Er plus αPD‐L1 treatments increased DC percentages from 1.4% for PBS to 2.3%, 2.7%, and 3.2%, respectively, and MHC II expression by 2.4‐, 2.8‐, and 5.1‐fold, respectively over PBS control (Figure 7c,d).

Tumor‐associated macrophages (TAMs) include antitumor M1 and protumor M2 phenotypes.^[^ ^43^ ^]^ While αPD‐L1 and Er‐NCP treatments only slightly increased M1 macrophage percentages in MC38 tumors, CuP, CuP/Er, and CuP/Er plus αPD‐L1 treatments significantly increased M1 macrophage percentages from 8.7% for PBS control to 16.7%, 20.1% and 29.7%, respectively (Figure 7e). CuP, Er‐NCP, CuP/Er, and CuP/Er plus αPD‐L1 treatments significantly reduced M2 macrophage percentages in MC38 tumors from 34.0% for PBS to 10.6%, 12.7%, 7.7%, and 3.7%, respectively (Figure 7f). Thus, CuP, Er‐NCP, CuP/Er, and CuP/Er plus αPD‐L1 treatments significantly increased M1/M2 ratios from 0.25 for PBS control to 1.58, 1.01, 2.61 and 8.03, respectively. The polarization of protumor M2 macrophages to antitumor M1 macrophages indicates strong antitumor innate immunity from CuP/Er and CuP/Er plus αPD‐L1 treatments.^[^ ^44^ ^]^

We also quantified intratumoral cytotoxic CD8^+^ T cells, helper CD4^+^ T cells, and immunosuppressive regulatory T cells (Tregs) after different treatments. While CuP/Er plus αPD‐L1 treatment significantly increased CD8^+^ T cell percentage to 4.1% from 1.6% for PBS control, the other treatments only slightly increased CD8^+^ T cell percentages (Figure 7g). Similarly, CuP/Er plus αPD‐L1 treatment significantly increased CD4^+^ T cell percentage to 5.6% from 0.7% for PBS control, the other treatments only slightly increased CD4^+^ T cell percentages (Figure 7h). Last, CuP/Er and CuP/Er plus αPD‐L1 treatments significantly reduced Treg percentages by 59.1% and 89.8%, respectively, from PBS control (Figure 7i).

We used enzyme‐linked immunospot (ELISpot) assay to detect tumor antigen‐specific, IFN‐γ secreting CD8^+^ T cells in the spleens.^[^ ^45^ ^]^ Splenocytes from treated MC38 tumor‐bearing mice were stimulated with KSPWFTTL (KSP) peptide and the IFN‐γ secreting spot‐forming cells (SFCs) were quantified. While αPD‐L1 and Er‐NCP treatments did not significantly increase the numbers of IFN‐γ secreting SFCs, CuP, CuP/Er, and CuP/Er plus αPD‐L1 treatments sufficiently increased IFN‐γ secreting SFCs per 10^6^ splenocytes from 0 for PBS to 97, 127, and 265, respectively (Figure 6c). These results demonstrate that CuP/Er plus αPD‐L1 significantly enhances antitumor efficacy by engaging both innate and adaptive immune responses.

The 4T1 tumor model is highly aggressive and easily metastasizes to different tissues.^[^ ^46^ ^]^ We established subcutaneous 4T1 tumor model on BALB/c mice. Seven days after tumor cell inoculation, mice with ≈100 mm^3^ tumors were randomized and i.v. injected with PBS, CuP, Er‐NCP, or CuP/Er at a dose of 4 mg kg^−1^ Cu or/and 35 mg kg^−1^ erastin once every three days for a total of three doses. A slightly higher Cu dose was used to elicit stronger antitumor effects. For αPD‐L1 groups, the mice were intraperitoneally injected with 75 ug per mice αPD‐L1 immediately after i.v. treatments. While αPD‐L1 treatment was ineffective with a TGI of 3.2%, CuP and Er‐NCP treatments moderately inhibited tumor growth with TGIs of 72.6%, and 52.2%, respectively. CuP/Er and CuP/Er plus αPD‐L1 treatments significantly increased the antitumor effects to afford TGIs of 92.3% and 99.1%, respectively (Figure 6b). Three out of six mice in the CuP/Er plus αPD‐L1 treatment group were tumor‐free, further supporting superb antitumor effects of the combination treatment.

Mice body weights were monitored daily, and mice did not show obvious weight losses in all treatment groups (Figure S29, Supporting Information). The hemolysis test showed that CuP/Er did not cause appreciable hemolysis at therapeutic doses (Figure S30, Supporting Information). Hearts, lungs, livers, spleens, and kidneys were harvested from treated mice and stained with haemotoxylin and eosin (H&E). Although no obvious histopathological abnormality was observed in these major organs, slight metastasis was observed in hearts and livers of control groups and significant metastasis was found in the lungs of control groups (Figure S32, Supporting Information). Pulmonary metastasis was evaluated by section analysis with cell detection and nucleus/cell ratio calculation. PBS, αPD‐L1, CuP, Era, CuP/Er, and CuP/Er plus αPD‐L1 groups showed tumor cell percentages among all pulmonary cells of 29.9%, 19.7%, 12.0%, 12.1%, 3.4%, and 1.8%, respectively (Figure 6d; Figure S32, Supporting Information). This result indicates significant reduction of pulmonary metastasis from CuP/Er and CuP/Er plus αPD‐L1 treatments.

We examined ICD induction by immunofluorescence analysis of CRT expression in tumor sections. CuP, Era, CuP/Er, and CuP/Er plus αPD‐L1 treatments significantly increased CRT signals in the tumors (Figure S22, Supporting Information). Interestingly, immunofluorescence showed that PD‐L1 expression in 4T1 tumors was greatly suppressed when αPD‐L1 was combined with CuP/Er.

Immunofluorescence studies also showed that CuP/Er plus αPD‐L1 treatment greatly increased CD11c and F4/80 signals, indicating increased DCs and macrophages in the tumors (Figure 6e). With enhanced induction of ICD and innate immune activation, CuP/Er plus αPD‐L1 significantly increased T cell populations in the tumors. The area ratios of CD3ε^+^CD4^+^/Hoechst and CD3ε^+^CD8^+^/Hoechst significantly increased from 0.34% and 0.19%, respectively, for PBS control to 2.43% and 2.74%, respectively, for CuP/Er plus αPD‐L1 treatment group (Figure 6f). Combination therapy of CuP/Er plus αPD‐L1 effectively inhibited tumor growth and tumor metastasis by engaging both innate and adaptive antitumor immunity without causing systemic toxicity.

Cu dysregulation has been observed in various human cancers,^[^ ^47^ ^]^ and over‐expression of Cu‐related proteins is correlated with the tumors' resistance to traditional platinum therapy.^[^ ^48^ ^]^ Although Cu chelators have been used to capture essential protein‐bound Cu to induce cell death,^[^ ^49^ ^]^ the utilization of Cu depletion in cancer therapy remains a challenge.^[^ ^50^ ^]^ However, the recent discovery of cuproptosis indicates that an excess amount of copper can also cause severe cell death and suggests a totally different strategy of exploiting Cu levels in cancer therapies.^[^ ^3^ ^]^ To this end, nanoparticles have been explored for cuproptosis‐based cancer treatment through tumor‐targeting and Cu accumulation in mitochondria.^[^ ^51^ ^]^

**Figure 8:**
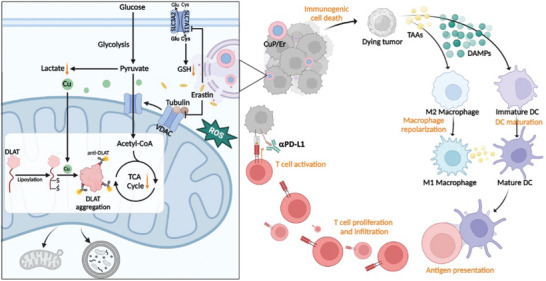
Scheme showing synergistic effects and immune activation by CuP/Er. Left, Er and Cu released from endocytosed CuP/Er induce ferroptosis, cuproptosis, and high ROS stress to cause cell death. Er reduces GSH levels by inhibiting the antiporter system Xc^−^ to sensitize cancer cells to cuprotosis. Er also opens VDACs on mitochondrial outer membranes to decrease glycolysis. Meanwhile, Cu directly binds to lipoylated TCA proteins (such as DLAT) to induce oligomerization and cause acute proteotoxic stress, which exhausts mitochondrial metabolism and leads to irreparable mitochondrial damage. Right, CuP/Er promotes DC maturation by inducing ICD and facilitates antigen presentation with released TAAs and DAMPs. CuP/Er also repolarizes macrophages from M2 to M1 phenotype, which further enhances antigen processing and presentation to naïve T cells. Primed T cells proliferate and infiltrate to the tumors while ICB by αPD‐L1 reinvigorates T cells for potent antitumor immunotherapy. The figure was generated with BioRender.

We have previously reported the tumor‐targeting ability of NCPs due to their long blood circulation and enhanced tumor uptake by targeting the over‐expressed receptors in tumors.^[^ ^52^ ^]^ Herein, we designed the core‐shell NCP CuP/Er for the co‐delivery of Cu, peroxide, and Er to tumor cells. As shown in **Figure** **8**, CuP/Er escapes from endo/lysosomes and releases Cu into the cytoplasm with the delivered peroxide. The co‐delivered Er triggers ferroptosis by increasing lipid peroxidation and depleting intracellular GSH. The opening of VDACs by Er decreases glycolysis and has anti‐Warburg effect, thus sensitizing cancer cells to CuP‐mediated cuproptosis. With gradual accumulation in the mitochondria, Cu‐mediated downregulation of essential active proteins like FDX1 and Cu binding to lipoylated TCA proteins (such as DLAT) and their oligomerization cause irreparable mitochondrion‐derived vesicles and mitophagy for severe cell death. CuP/Er releases DAMPs like CRT, HMGB‐1, and ATP, which in combination with tumor‐associated antigens (TAAs), promote APC maturation and antigen presentation to T cells. As CuP‐mediated cuproptosis up‐regulates PD‐L1 expression in cancer cells, CuP/Er‐mediated ferroptosis and cuproptosis synergize with ICB to elicit potent antitumor immunity, leading to regression of MC38 and 4T1 tumors and prevention of lung metastasis of 4T1 tumors. The observation of mitochondrion‐derived vesicles and mitophagy suggests the possibility of combining cuproptosis with autophagy inhibitors to further enhance treatment effects by complete destroying mitochondria and preventing cancer cells from recovering their functions.^[^ ^53^ ^]^

## 3 Conclusion

In this work, we demonstrate a new strategy to enhance cancer treatment by synergizing ferroptosis and cuproptosis using NCP particles. CuP/Er sensitizes tumor cells to cuproptosis by reducing aerobic glycolysis and inhibits the TCA cycle via oligomerization of lipoylated TCA proteins in the mitochondria. Consequently, CuP/Er depletes GSH, enhances lipid peroxidation, and causes irreparable mitochondrial damage to inhibit the growth of breast and colon tumors in mouse models. Further combination of CuP/Er with an anti‐PD‐L1 antibody (αPD‐L1) potently regresses tumors and prevents tumor metastasis via synergizing ICD by CuP/Er and reinvigoration of T cells by ICB.^[^ ^54^, ^55^, ^56^ ^]^ Our findings suggest the potential of utilizing Cu ions as an anticancer agent to overcome resistance to conventional cancer therapy. The combination of NCP particles with ICB shows promise as a new strategy to potentiate cancer immunotherapy.

## Conflict of Interest

W.L. is the founder of Coordination Pharmaceuticals, Inc., which licenses the NCP technology from the University of Chicago. R.R.W. is a scientific advisor to Coordination Pharmaceuticals. All other authors declare no competing financial interest.

## Supporting information

Supporting Information

Supplemental Movie 1

Supplemental Movie 2

## Data Availability

The data that support the findings of this study are available from the corresponding author upon reasonable request.
