# Supplementary material for: Nanoparticles Synergize Ferroptosis and Cuproptosis to Potentiate Cancer Immunotherapy
Source: Adv Sci (Weinh). 2024 Mar 13;11(23):2310309. doi: 10.1002/advs.202310309 (PMC11187894; doi:10.1002/advs.202310309)
Supplement: Supplementary file 1 — Supporting Information [file ADVS-11-2310309-s003.pdf]

ADVANCED SCIENCE
Open Access

Supporting Information

for *Adv. Sci.*, DOI 10.1002/advs.202310309

Nanoparticles Synergize Ferroptosis and Cuproptosis to Potentiate Cancer Immunotherapy

*Youyou Li, Jing Liu, Yimei Chen, Ralph R. Weichselbaum and Wenbin Lin**

Supporting Information

Nanoparticles Synergize Ferroptosis and Cuproptosis to Potentiate Cancer Immunotherapy

Youyou Li, Jing Liu, Yimei Chen, Ralph R. Weichselbaum, and Wenbin Lin *
E-mail: wenbinlin@uchicago.edu

1. Experimental Section

Materials and Instruments. All starting materials were purchased from Sigma-Aldrich and Fisher (USA), unless otherwise noted, and used without further purification. Transmission electron microscopy (TEM) was carried out on a TECNAI Spirit instrument. Particle sizes were measured via dynamic light scattering (DLS) on a Malvern Nano Series Zeta-Sizer. Flow cytometry data was collected on an LSR-Fortessa 4-15 (BD Biosciences) and analyzed by FlowJo software. Confocal laser scanning microscope images were collected on Leica SP8 and analyzed with ImageJ software. ICP-MS results were obtained on a 7700 series ICP-MS (Agilent Technologies).

Cell Lines and Animals. Murine colon adenocarcinoma MC38 cells and murine metastatic triple-negative breast carcinoma 4T1 cells were purchased from the American Type Culture Collection (Rockville, MD, USA) and cultured in DMEM medium (GE Healthcare, USA) or RPMI-1640 (Corning, USA) supplemented with 10% fetal bovine serum, 100 U/mL penicillin G sodium and 100 μg/mL streptomycin sulfate in a humidified atmosphere containing 5% CO2 at 37 ºC. C57BL/6 mice (female, 6 weeks, 18-22 g) and BALB/c female mice (6 weeks, 18-22 g) were provided by Harlan Laboratories, Inc (USA). The study protocol was reviewed and approved by the Institutional Animal Care and Use Committee (IACUC) at the University of Chicago. The Human Tissue Resource Center at the University of Chicago provided histology related services for this study.

Synthesis of CuP bare particle. An aqueous mixture of Na_3PO_4 (100 μL, 5mg/ml) and 1,2-dioleoyl-sn-glycero-3-phosphate (DOPA) (150 μL, 200 mg/mL) in a 5 mL surfactant system (0.3 M Triton X-100 and 1.5 M 1-hexanol in cyclohexane) was sonicated for 1 min. Another aqueous solution of $CuCl_2$ (100 μL, 300 mg/mL) and 30% H_2O_2 (100 μL) in 5 mL of the same surfactant system was stirred vigorously. The $CuCl_2$/H_2O_2 solution was added dropwise to the Na_3PO_4/DOPA solution and stirred at room temperature for 10 min. Thirty milliliters of ethanol were added. DOPA-capped CuP bare particles were obtained by centrifugation at 12,000 ×g at 4 °C for 5 min.

The resulting particles were washed with ethanol three times and redispersed in tetrahydrofuran for lipid coating. The final copper concentration was quantified by ICP-MS.

X-ray photoelectron spectroscopy (XPS) analysis. Survey and high-resolution O 1s and Cu 2p XPS spectra of CuP bare particle was obtained on a Kratos AXIS Nova instrument with a monochomatic Al Kα X-ray source and a delay line detector system. Collected data was calibrated by C 1s at 284.8 eV and fitted with the CasaXPS software.

Synthesis of CuP, Er-NCP and CuP/Er. For the preparation of CuP, a THF solution of CuP-bare particle, DSPE-PEG2K (300 µL, 5 mg/mL), DOPC (420 µL, 5 mg/mL), and cholesterol (84 µL, 5 mg/mL) in THF solution were added dropwise to 1 mL of 30% ethanol/water and vigorously stirred. The organic solvents (THF and ethanol) were completely evaporated under a nitrogen flow, and the resultant CuP particle was diluted in 1.0 mL phosphate-buffered saline (PBS) for further use. CuP/Er was similarly prepared by mixing CuP-bare particle with DSPE-PEG2K, DOPC, erastin, and cholesterol during shell coating. Er-NCP was prepared by replacing CuP-bare particle with ZnP-bare particle.[1]

Synthesis of Ce6-NCP. Ce6-NCP was prepared by adding a microemulsion of Ce6 (100 µL, 5mg/ml) to a microemulsion of $Zn(NO_3)_2$ (100 µL, 600 mg/mL) to obtain the bare particle, and then coated with DSPE-PEG2K, DOPC and cholesterol.

Copper release from CuP/Er. CuP/Er in 3.5k MWCO dialysis cup (Thermo Fisher Scientific) was immersed in 50 ml PBS (pH=7.4 or 5.5) at 37 °C. At pre-set time intervals, 100 µL of sample was withdrawn and the Cu concentration in each aliquot was quantified using ICP-MS. Cu release percentage was calculated as the total amount of Cu in PBS solution / total amount of Cu in the added CuP/Er.

Colorimetric titration assay and GC analysis. CuP was titrated with 50 µg/ml $KMnO_4$ in 0.1 M H_2SO_4 solution. The amount of O-O bond in CuP was calculated by the following equation: $2MnO_4^- + 5(\text{-O-O-}) + 16H^+ \rightarrow 2Mn^{2+} + 5O_2 + 8H_2O$. The amount of O_2 generated after the addition of excess $KMnO_4$ was also quantified by gas chromatography with an Agilent 7890B GC system.

Hydrogen peroxide, hydroxyl radical and total ROS generation in test tubes. 50 µM CuP/Er in 100 µl PBS (pH=7.4 or 5.5) in a black 96-well plate was added Hydrogen Peroxide Assay Kit (Sigma-Aldrich) for hydrogen peroxide, APF kit (Thermo Fisher Scientific) for hydroxyl radical or H_2DCFDA kit (Thermo Fisher Scientific) for total ROS determination. The fluorescence (exiting: 485/20 nm; emission: 520/20 nm) was read immediately by Synergy HTX.

Cellular uptake. 5×10^5 4T1 cells were seeded in 6-well plates and incubated with 1 μM CuP/Er for different lengths of time. The cells were collected and washed with PBS three times. Cell numbers were counted before digestion by 70% HNO_3 for Cu analysis by ICP-MS.

Subcellular localization. 10^5 4T1 cells were seeded in dishes and incubated with Ce6-NCP for different lengths of time. After washing with PBS three times, the cells were stained with 100 nM LysoTracker™ Green DND-26 (Thermo Fisher Scientific) for 15 min and then washed with PBS three times again before imaging under CLSM.

Mitochondrial Cu content determination. 4T1 cells were seeded in 6-well plates and 24 hours later, 1 μM CuP/Er was added. After different time intervals, the cells were collected and counted. The mitochondria were extracted by Mitochondria Isolation Kit (Thermo Fisher Scientific), then Cu content was determined by ICP-MS.

Hydrogen peroxide, hydroxyl radical and total ROS generation in vitro. 10^5 4T1 cells were seeded in 6-well plates and incubated with 2 μM CuP, Er-NCP, or CuP/Er for 24 h. The cells were washed with PBS three times and stained with Hydrogen Peroxide Assay Kit (Sigma-Aldrich) for hydrogen peroxide, APF kit (Thermo Fisher Scientific) for hydroxyl radical or H_2DCFDA kit (Thermo Fisher Scientific) for total ROS with optimized sensor concentrations and staining time. After washing with PBS three times, the stained cells were immediately harvested for flow cytometry.

Lysosomal escape. 10^5 4T1 cells were seeded in dishes and incubated with 2 μM CuP, Er-NCP or CuP/Er containing Ce6 in the core for 8 h. After washed with PBS three times, the cells were stained with 100 nM LysoTracker™ Green DND-26 for 15 min. The cells were washed with PBS three times and immediately observed by CLSM.

Lipid peroxidation. 10^5 4T1 cells were seeded in dishes and incubated with 2 μM CuP, Er-NCP, or CuP/Er for 24 h. After washing with PBS three times, the cells were stained with BODIPY™ 581/591 C11 (Thermo Fisher Scientific) for 15 min. The cells were washed with PBS three times and immediately observed by CLSM or harvested for flow cytometry.

SLC7A11 Expression. 2×10^5 4T1 cells were seeded in 6-well plates and incubated with 2 μM CuP, Er-NCP, or CuP/Er for 24 h. The cells were collected and washed with PBS three times. The cells were fixed with 4% paraformaldehyde (PFA) for 10 minutes and then blocked and permeabilized by 5% FBS + 0.2% Triton-X in PBS for 15 min. After permeabilization, the cells were incubated with anti-mouse SLC7A11 antibody (Cell signaling) in 1% BSA at 4 °C overnight. The cells were then washed with PBS three times and incubated with secondary antibodies (AF647

anti-rabbit IgG, Cell signaling) in 1% BSA at room temperature for 1 hour. The cells were washed three times and collected for flow cytometry.

GSH and GSSG analysis. 2×10^5 4T1 cells were seeded in 6-well plates and incubated with 2 μM CuP, Er-NCP or CuP/Er for 24 h. The media were discarded, and the cells were washed with PBS. Then, the cells were lysed and analyzed with the GSH/GSSG assay kit (Sigma-Aldrich).

Mitochondrial membrane potential change. 10^5 4T1 cells were seeded in dishes and incubated with 2 μM CuP, Er-NCP or CuP/Er for 24 h. After washing with PBS three times, the cells were stained with 10 μM JC-1 for 15 min. Then, the cells were washed with PBS three times to remove excess JC-1 and immediately observed by CLSM or collected for flow cytometry.

DLAT Foci analysis. 10^5 4T1cells were seeded in dishes and incubated with 2 μM CuP, Er-NCP or CuP/Er for 24 h. The cells were collected and washed with PBS three times. The cells were fixed with 4% PFA, and blocked and permeabilized by 5% FBS + 0.2% Triton-X in PBS. The cells were then incubated with DLAT antibody (13426-1-AP, Thermo Fisher Scientific) in 1% BSA at 4 °C overnight. The cells were washed with PBS three times and incubated with secondary antibodies (AF647 anti-rabbit IgG, Cell signaling) in 1% BSA at room temperature for 1 hour. The cells were washed with PBS three times and stained by MitoTracker™ Red CMXRos (Thermo Fisher Scientific) and Hoechst (Thermo Fisher Scientific). Foci counter[2] (automatic background was used and tolerance setting was 75[3]) plugin was used to mask foci segmentation and counted the numbers of foci. The final results were normalized to foci count per cell in each well. The Z-stack images were reconstructed into 3D images by 3D viewer plugin[4] after foci were masked by foci counter plugin.

TEM imaging of 4T1 cells. 4T1 cells were treated with 2 μM CuP, Er-NCP, or CuP/Er for 24 h. The cells were fixed with 4% PFA and 2% glutaraldehyde in 0.1 M sodium cacodylate at 4 °C overnight. The cells were subsequently post-fixed with 1% osmium tetroxide for 1 h and 1% uranyl acetate buffer for 1 h. Dehydration was performed using 25, 50, 70, 95 and 100 ethanol, and 100% propylene oxide sequentially and embedded in gradually increasing Spurr resin:propylene oxide mixed solution (1:2 to 1:1) and finally 100% Spurr resin. Following this, the cells were cut into 90 nm ultrathin sections with Leica EM UC60, placed on a copper grid and stained with uranyl acetate and led citrate. Observations and photographs were obtained using FEI Tecnai F30 and Gatan CCD digital micrograph.

Cytotoxicity. 4T1 cells or HEK293T cells were seeded in a 96-well plate (2×10^3 cells/well) and incubated with different concentration of the NCP carrier, CuP, Er-NCP, or CuP/Er for 48 h. Cell viability was determined by MTS assay.

CRT, HSP70 and PD-L1 expression. For flow cytometry, $2×10^5$ 4T1cells were seeded in 6-well plates and incubated with 2 μM CuP, Er-NCP, or CuP/Er for 24 h. The cells were harvested and then stained with different antibodies: CRT (AF488 calreticulin antibody; Novus bio), HSP70 (AF647 HSP70 antibody; Novus bio) or PD-L1 (APC anti-mouse PD-L1antibody; Biolegend) before analysis by flow cytometry. For CRT confocal experiments, the cells after treatments were fixed with 4% PFA, and then stained with CRT antibody in 1% BSA at 4 °C overnight. The cells were washed three times before observation under CLSM.

HMGB1 release, ADP/ATP ratio, and L-Lactate quantification. 4T1 cells were seeded in 6-well plates and incubated with 2 μM CuP, Er-NCP or CuP/Er for 24 h. The media or cells were collected by centrifugation, and tested by HMGB1 Detection kit (Chondrex), ADP/ATP Ratio Assay Kit (Sigma-Aldrich) or L-Lactate Assay Kit (Cayman Chemical).

Western blot. 4T1 cells were seeded in 6-well plates and incubated with 2 μM CuP, Er-NCP, or CuP/Er for 24 h. The cells were collected, lysed with RIPA in the presence of a protease inhibitor (Thermo Fisher Scientific). The protein was quantified by BCA kit (Thermo Fisher Scientific) and separated by 4–12% NuPAGE Bis-Tris Mini Gels (Thermo Fisher Scientific). After transfer, the membranes were blocked, then incubated with primary antibodies against FDX1 (EPR4629, Abcam) or GPX4 (PA5-120674, Thermo Fisher Scientific), and secondary antibody Anti-rabbit IgG, HRP-linked Antibody (Cell Signaling Technology), and ECL Western Blotting Substrate (Thermo Fisher Scientific), and finally exposed under the FluorChem R system (ProteinSimple).

Mechanistic study with different inhibitors. 4T1 cells were seeded in 6-well plates and 6 hours later, 10 μM ferrostatin 1 (Fer-1), 10 μM UK5099, or 30 μM Z-VAD-FMK was added. After incubation for 16 hours, 2 μM CuP/Er was added. ROS generation, lipid peroxidation, cell death, and DAMPs release were tested as above.

Antigen presentation. Bone marrow-derived dendritic cells (BMDCs) were obtained from the marrow cavities of 6-week-old female C57BL/6 mice. After extraction BMDCs were cultured in RPMI-1640 medium containing GM-CSF (20 ng/mL) and IL-4 (10 ng/mL). The purify of BMDCS was checked by flow cytometry. 4T1 cells were incubated with 2 μM CuP, Er-NCP or CuP/Er for 24 h. The media were then removed. Treated 4T1 cells were co-cultured with BMDCs for 24 h. The BMDCs were collected and blocked by anti-mouse CD16/CD32 antibody (eBioscience) and stained with mouse BV421 anti-CD45, FITC anti-CD11b, PE-EF610 anti-CD11c, PE-Cy7 anti-CD80, APC anti-CD86 and PE anti-MHC II. The percentage of matured DCs and the expression of MHC II of BMDCs were detected by flow cytometry.

***In vivo* antitumor efficacy.** MC38 cells (2×10^6) or 4T1 cells (1×10^6) were subcutaneously injected into the right flank regions of 6-week-old C57BL/6 mice. Seven days later, the tumor-

bearing mice with average tumor volume of 100 mm^3 were randomly grouped (n = 6) and intravenously injected with PBS, CuP, Er-NCP, or CuP/Er at an equivalent dose of 3.5 mg/kg Cu or/and 30 mg/kg erastin (MC38 model) or 4 mg/kg Cu or/and 35 mg/kg erastin (4T1 model) once every 3 days (Q3D) for 3 doses. 75 µg αPD-L1 (BioXCell) was intraperitoneally injected followed each i.v. injection. Tumor volumes (width × length × width)/2 were monitored. TGI was calculated as (1-average of tumor volumes of treated group/ average of tumor volumes of control group)×100%.

Mouse body weights were monitored. Mice were sacrificed when the tumors of the control group reached 2 cm^3. Excised 4T1 tumors were photographed, and cryo-sectioned for immunofluorescence staining. Hearts, lungs, livers, kidneys, and spleens were harvested and sectioned for H&E staining for systemic toxicity evaluation. Metastatic tumors were found in lungs when the mice were dissected. Lungs H&E slides were analyzed by QuPath software for evaluating tumor metastasis after different treatments.

Immune cell profiling. MC38 tumor-bearing C57BL/6 mice were treated as in antitumor efficacy experiments. On day 12 post the first i.v. injection, tumors and tumor-draining lymph nodes (TDLNs) were harvested for immune cell profiling by flow cytometry. Briefly, tumors and lymph nodes were digested with a cocktail with 1 mg/mL collagenase I + 1 mg/mL collagenase IV + 1 mg/mL DNase I in complete medium at 37 °C for 30 min. The tissues were ground and filtered through sterile cell strainers to complete medium to obtain single cell suspensions. The cells were obtained by centrifugation (300 g, 5 min) at 4 °C and stained with LIVE/DEAD™ fixable yellow dead cell stain kit (ThermoFisher Scientific), then blocked with mouse anti-CD16/32 antibody at 4 °C for 5 min. The lymph node cells were stained with the following fluorochrome conjugated anti-mouse antibodies at 4 °C for 30 min: BV421 anti-CD45, FITC anti-CD11b, PE-EF610 anti-CD11c, PE-Cy7 anti-CD80 and APC anti-CD86. Tumor cells were stained by BV421 anti-CD45, FITC anti-CD11b, PE-EF610 anti-CD11c, PerCP-Cy5.5 anti-F4/80, APC anti-CD86, Pe-Cy7 anti-CD206 and PE anti-MHC II (for DCs and macrophages profiling) or BV421 anti-CD45, PE-EF610 anti-CD3ε, APC-Cy7 anti-CD4, PerCP-Cy5.5 anti-CD8 and APC anti- Foxp3 (for T cells profiling). All antibodies were obtained from eBioscience except those specifically designated. Before Foxp3 staining, the cells were treated with Foxp3/Transcription Factor Staining Buffer Set (eBioscience). Compensation was calculated and results were analyzed by FlowJo software.

IFN-γ ELISPOT Assay. A MultiScreen HTS sterile 96 well filter plate (MilliporeSigma) was activated with 35% ethanol for 1 min, washed with water, coated with capture antibody (IFN gamma Mouse Kit, Thermo Fisher Scientific) at 4 °C overnight, and then blocked with 10% FBS at room temperature for 2 h. Spleens harvested from the treated MC38 tumor-bearing C57BL/6 mice were ground and filtered through sterile cell strainers to afford single-cell splenocyte suspensions. Red blood cells were lysed by ACK lysis buffer (Thermo Fisher Scientific), and

splenocytes were counted and seeded in the plates at the same density of 10^6 cells/well in RPMI-1640 complete medium. 10 μg/mL KSPWFTTL (KSP) peptide was added to each well except for negative control. The splenocytes in positive control wells were stimulated with mouse anti-CD3ε and anti-CD28 antibody (2 ug/ml, eBioscience). The plate was incubated at 37 °C for 48 h. After washing, biotinylated detection antibody was added and incubated at 37 °C for 2 h. Excess detection antibodies were washed away and streptavidin-HRP conjugate was added. Finally, AEC substrate (BD Biosciences) was added for spot forming. The plate was totally dried in the dark and spot pictures and numbers were taken and analyzed by CTL ImmunoSpot S6.

Tumor metastasis evaluation. The whole lung slides were analyzed by QuPath software after automated detection of cell nuclei and cell borders. Nucleus/cell ratio was calculated for every single cell and the same threshold considered as tumor cells was set for all slides. The percentage of tumor cells number over all lung cells number was calculated as tumor metastasis percentage in the lungs of 4T1 tumor-bearing mice after different treatments.

Immunofluorescence staining of cryo-sectioned slides. 4T1 tumors were harvested and frozen at -80 °C freshly with OCT compound (Fisher Healthcare). The blocks were sectioned by the Human Tissue Resource Center. The slides were fixed with 75% acetone + 25% ethanol, blocked with 10% FBS, and then incubated respectively with mouse AF488 anti-CD4, APC-EF780 anti-CD8, PE-EF610 anti-CD3ε, APC anti-PD-L1, CRT antibody, APC anti-F4/80, or PE-EF610 anti-CD11c at a 4 °C overnight before observation by CLSM.

Hemolysis assay. Fresh mouse blood was washed with PBS several times to obtain erythrocytes. Then, 5% erythrocytes in PBS (negative control), in PBS with different concentrations of CuP/Er, and in 0.1% Trion-X-100 aqueous solution (positive control) were incubated at 37 °C for 1 hour. The samples were centrifuged, and the absorbances of the supernatants (OD) were measured at 540 nm. Hemolysis rates were calculated as $(OD_{sample}-OD_{negtive})/(OD_{positive}-OD_{negtive})\times100\%$.

Statistical analysis. All the statistical analysis was performed by One-way Repeated Measures ANOVA method with Tukey's honest significance test to determine whether the difference between each group was significant. The p values were defined as * $p<0.05$, ** $p<0.01$, *** $p<0.001$ in all the figures.

2. Supporting Figures

a

Particle	Core					Shell	
	Cu	~O-O~	Ce6	Zn	DOPA	Erastin	Cholesterol DOPC DSPE-PEG2K
CuP-bare	Yes	Yes			Yes		
CuP	Yes	Yes			Yes		Yes
Er-NCP				Yes	Yes	Yes	Yes
CuP/Er	Yes	Yes			Yes	Yes	Yes
Ce6-NCP			Yes	Yes	Yes		Yes
CuP-Ce6/Er	Yes	Yes	Yes		Yes	Yes	Yes

b

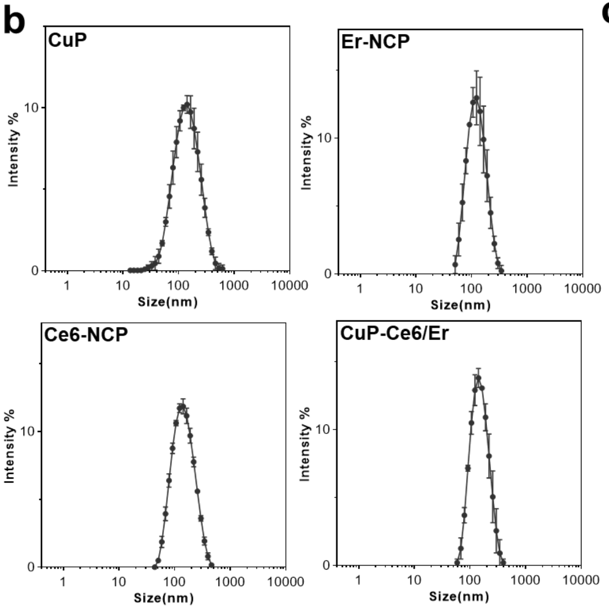

c

Particle	Size (nm)	PDI
CuP-bare	78.4 ± 0.6	0.19 ± 0.01
CuP	131.7± 1.7	0.32 ± 0.09
Er-NCP	138.7 ± 1.7	0.27 ± 0.04
CuP/Er	146.8 ± 1.6	0.24 ± 0.03
Ce6-NCP	133.4 ± 1.5	0.23 ± 0.05
CuP-Ce6/Er	158.7 ± 1.9	0.32 ± 0.07

Figure S1. (a) Composition of particles used in this research. (b,c) Size distribution of particles by DLS. All data are represented as mean ± SD, n = 3.

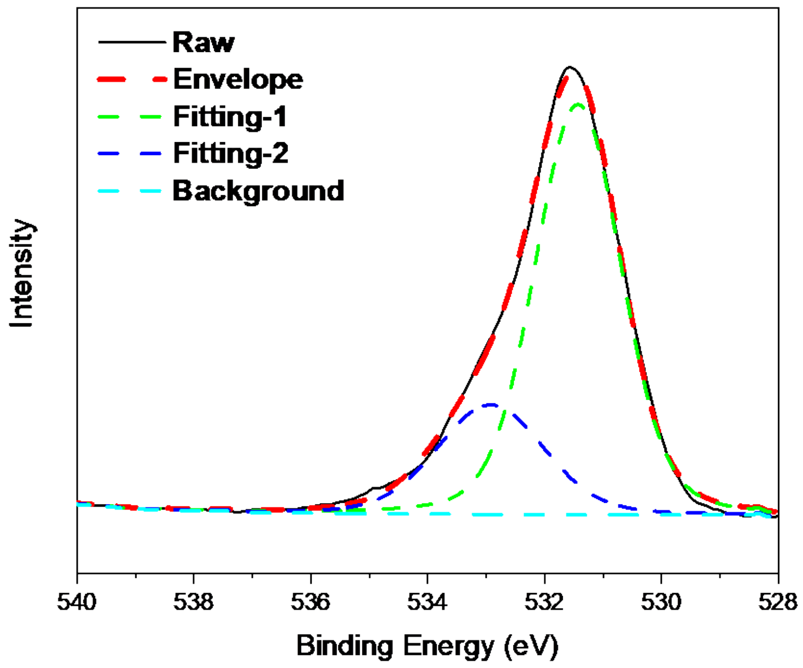

Figure S2. Fitting of O 1s XPS spectra of CuP bare particle.

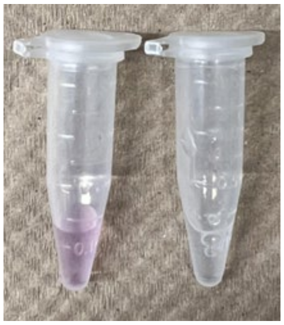

Figure S3. Color change of 50 μg/ml $KMnO_4$ in 0.1 M H_2SO_4 solution tiltrated by NCP CuP.

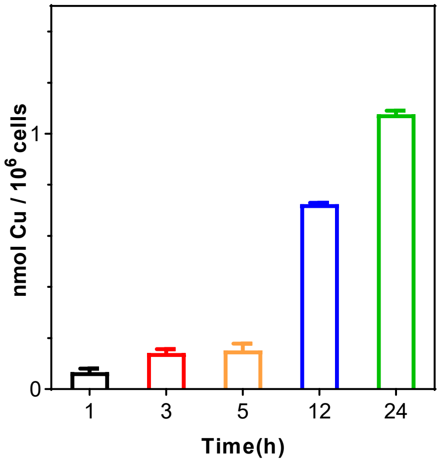

Figure S4. Uptake of CuP/Er by 4T1 cells after incubation for different lengths of time.

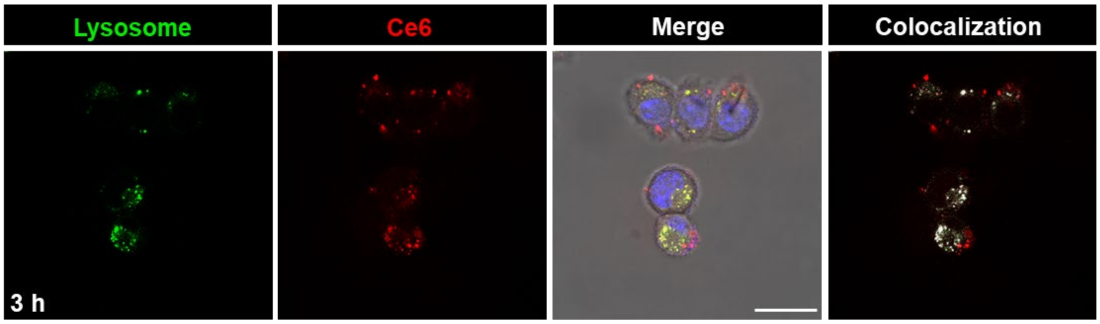

Figure S5. Lysosome and Ce6 colocalization after incubating 4T1 cells with Ce6-NCP for 3 hours.

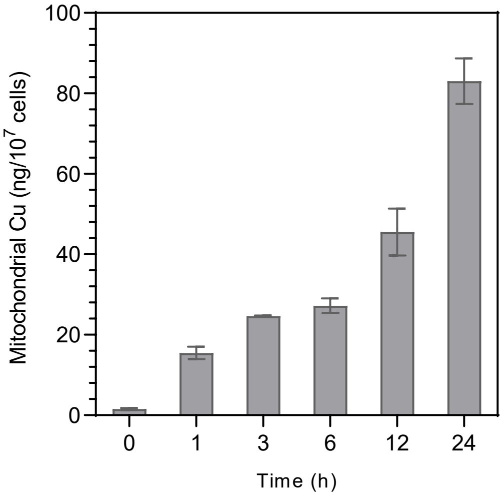

Figure S6. The Cu contents in the mitochondria of 4T1 cells after CuP/Er treatment for different lengths of time.

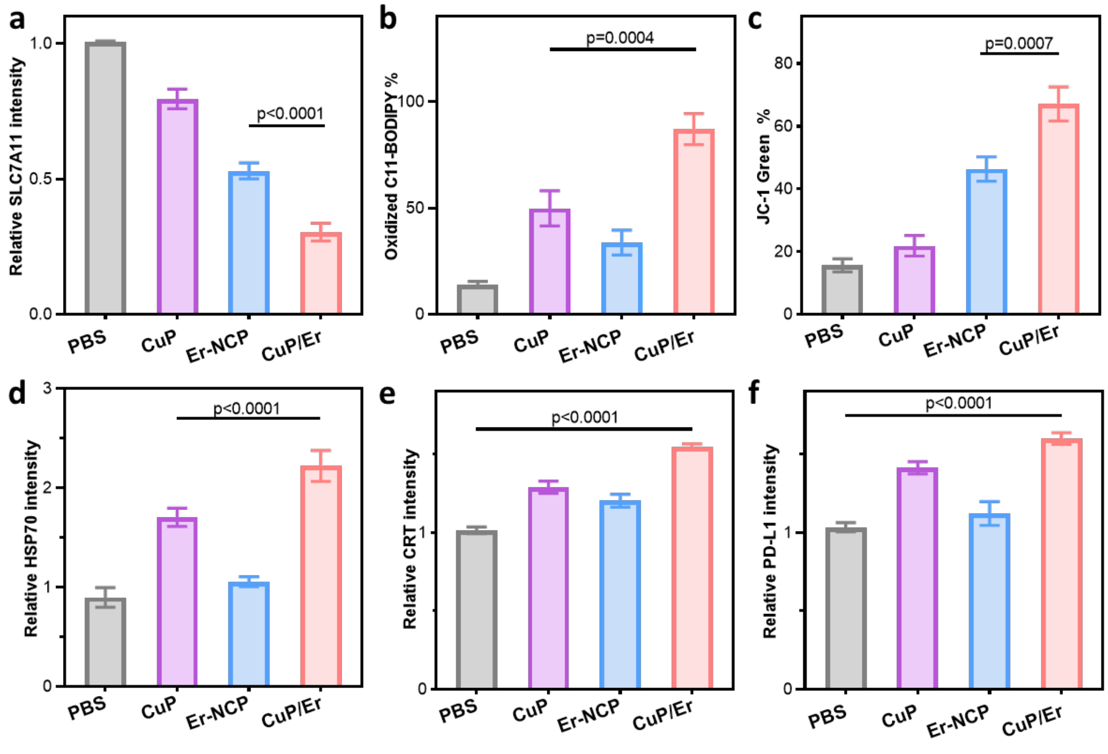

Figure S7. (a) Relative intensity of SLC7A11 by flow cytometry, (b) Lipid peroxidation representing by Oxidized C11-BODIPY percentage in **Figure 3g**, (c) Mitochondrial membrane potential change represented by JC-1 green percentage in **Figure 3h**, (d) Relative intensity of HSP70 by flow cytometry, (e) Relative intensity of CRT by flow cytometry and (f) Relative intensity of PD-L1 by flow cytometry in tumor cells treated with different NCPs. All data are represented as mean ± SD, n = 3.

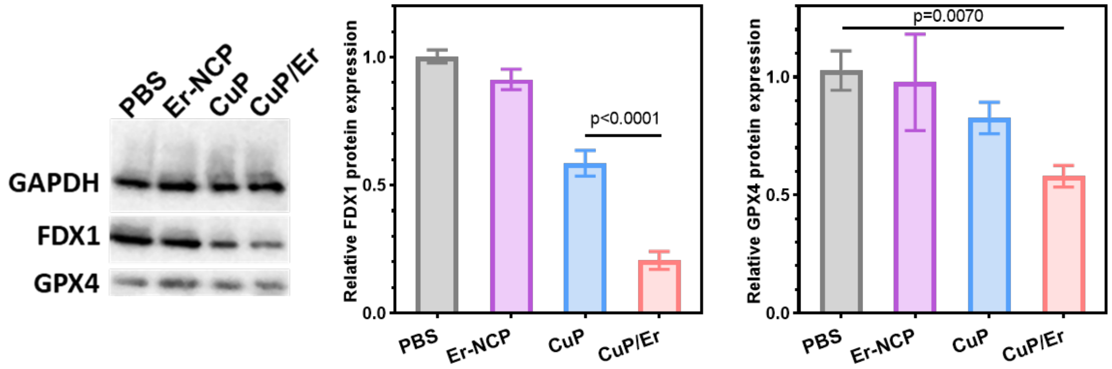

Figure S8. Western blot results showing FDX1 and GPX4 expressions of 4T1 cells treated with 2 μM CuP, Er-NCP, or CuP/Er for 24 h.

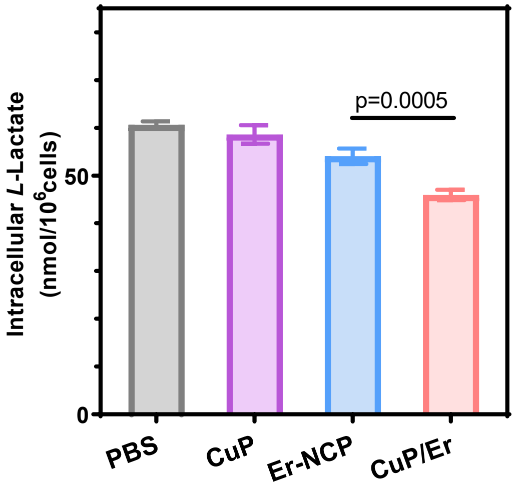

Figure S9. Intracellular L-lactate of tumor cells treated with different NCPs. Data are represented as mean ± SD, n = 3.

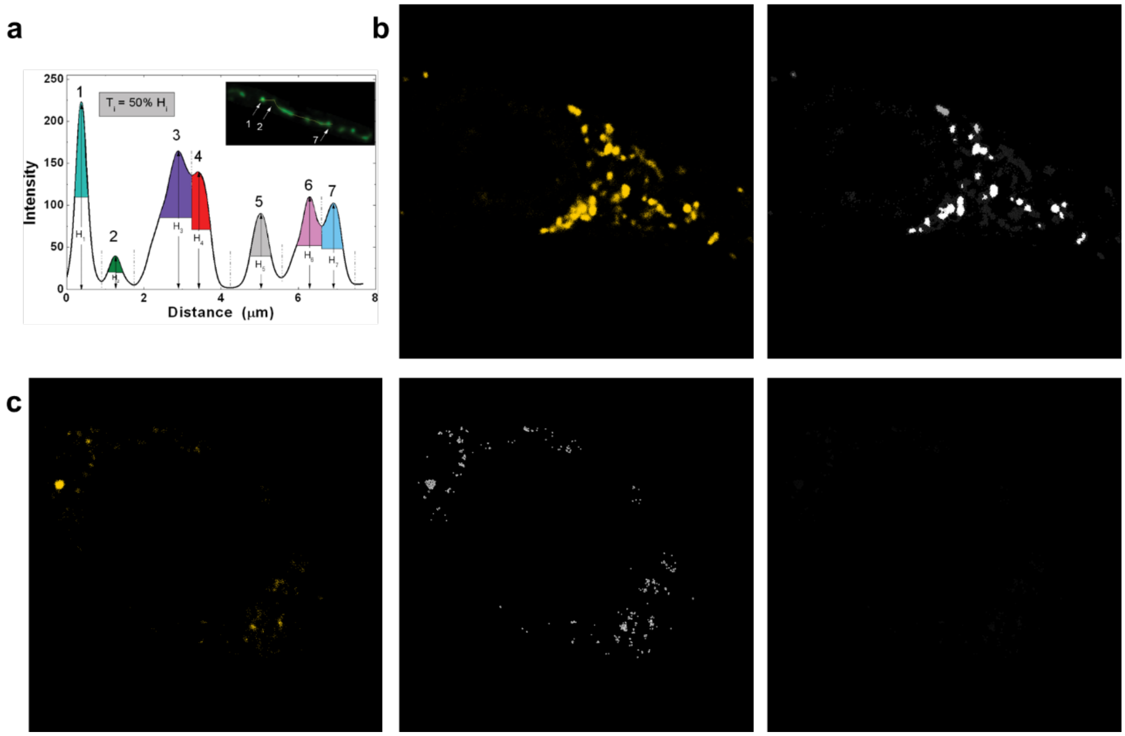

Figure S10. (a)Representative foci recognition process by foci mask.[2] (b) Representative DLAT signals recognized as foci and (c) DLAT signals not recognized as foci.

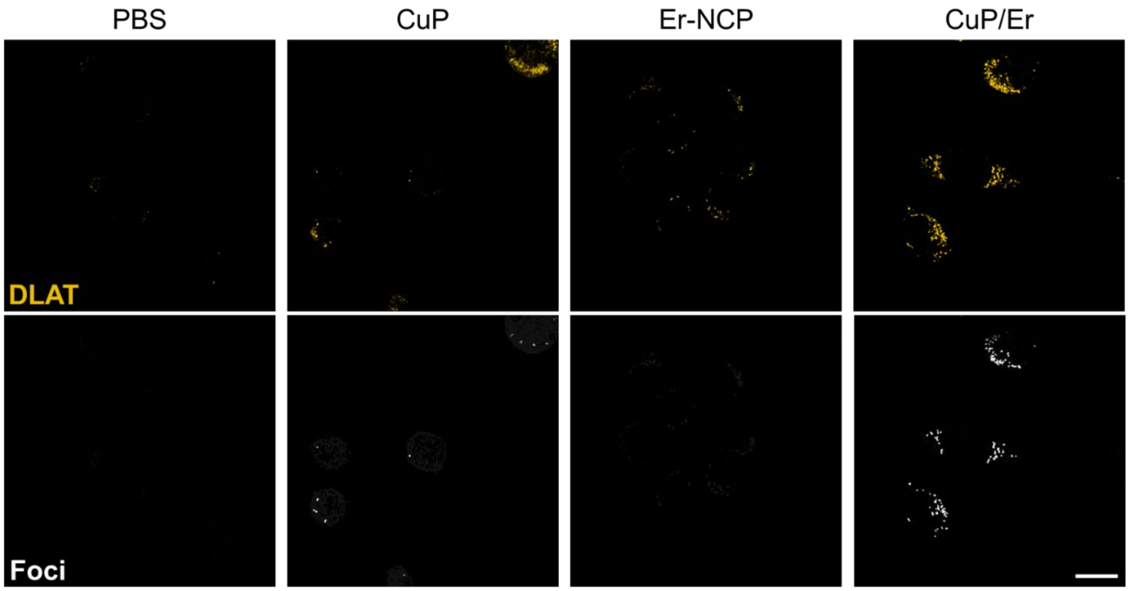

Figure S11. Images of separated DLAT channels and Foci masks of DLAT channels in Figure 4a.

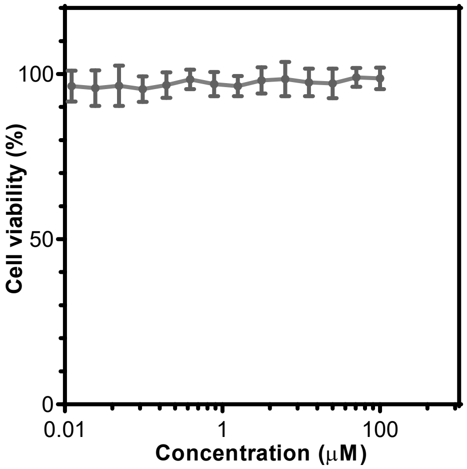

Figure S12. Viability 4T1 cells after treatment with the NCP carrier. The X-axis refers to Zn concentration. All data are represented as mean ± SD, n = 3.

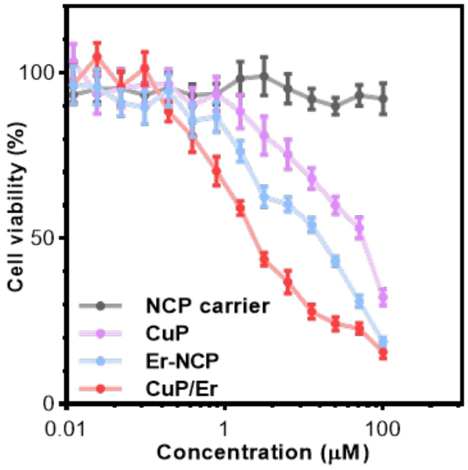

	IC_{50} (μM)
CuP	44.1 ± 10.5
Er-NCP	12.4 ± 2.6
CuP/Er	3.3 ± 0.6

Figure S13. Viability of HEK293T cells with different treatments. For the NCP carrier, the X-axis refers to Zn concentration. All data are represented as mean ± SD, n = 3.

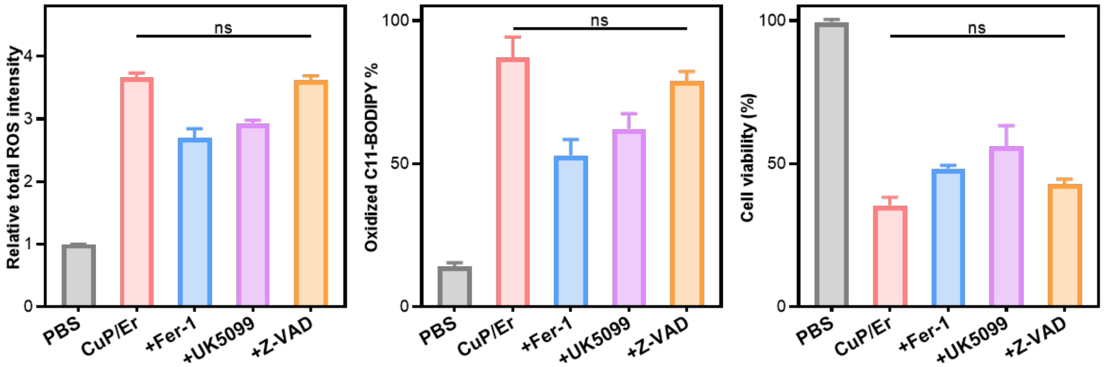

Figure S14. ROS generation and lipid peroxidation in and viability of CuP/Er-treated 4T1 cells in the absence or presence of 10 μM ferrostatin 1 (Fer-1), 10 μM UK5099, or 30 μM Z-VAD-FMK.

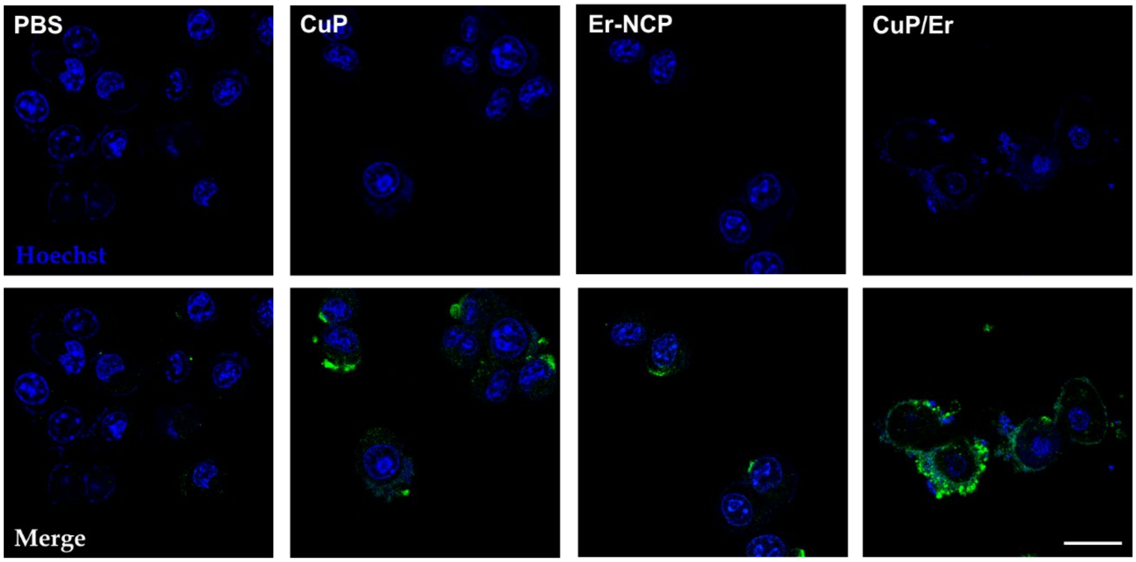

Figure S15. Confocal images of CRT expression in 4T1 cells after different treatments (shown as nucleus staining and merged images as supplements for Figure 5a). Scale bar is 20 μm.

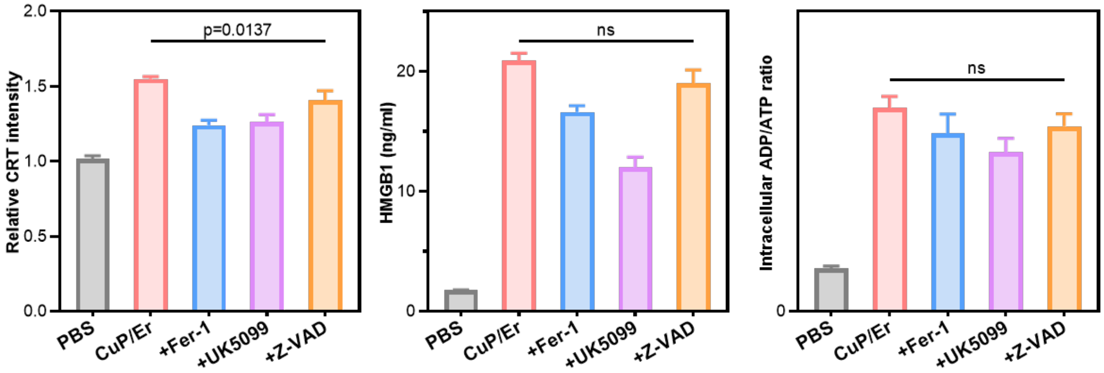

Figure S16. CRT expression, HMGB1 concentrations and intracellular ADP/ATP ratios in CuP/Er-treated 4T1 cells with or without the addition of 10 μM Fer-1, 10 μM UK5099, or 30 μM Z-VAD-FMK.

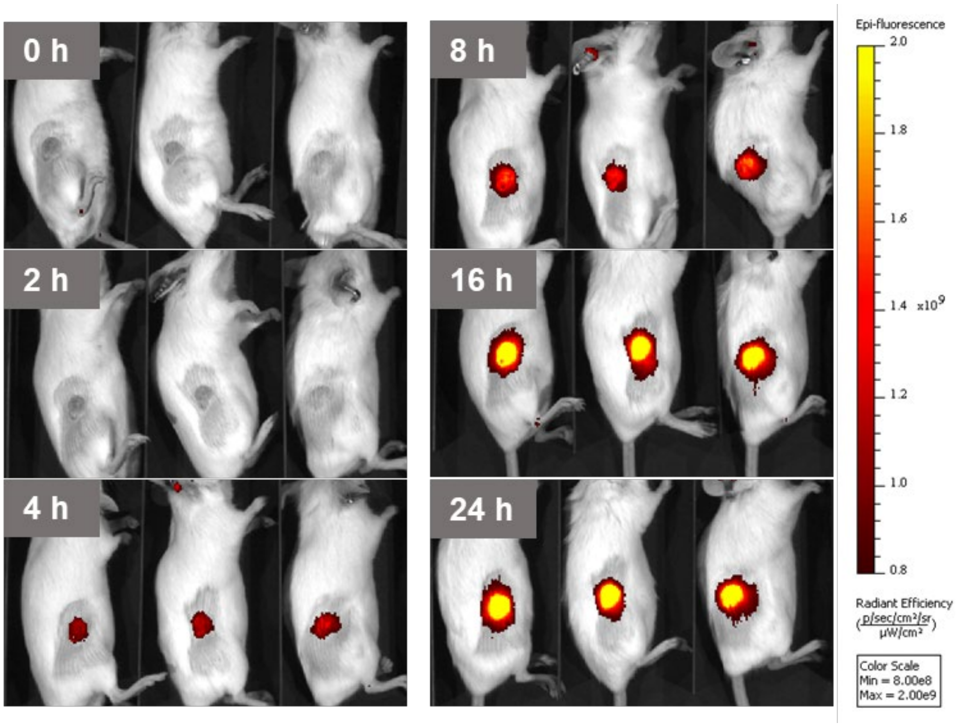

Figure S17. *In vivo* imaging of 4T1 tumor-bearing mice after CuP-Ce6/Er intravenous injection over 24 hours.

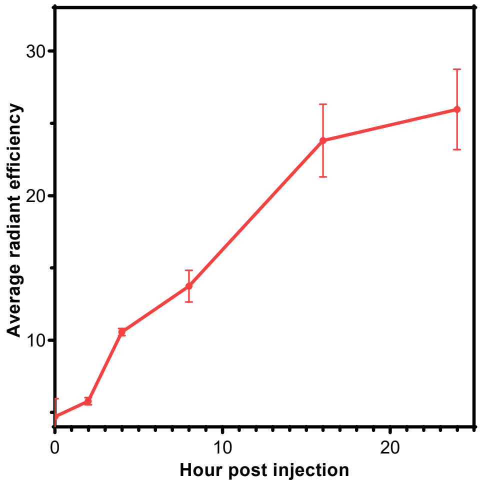

Figure S18. Tumors average radiant efficiency of 4T1 tumor-bearing mice after CuP-Ce6/Er intravenous injection. Data are represented as mean ± SD, n = 3. The unit is ($\times 10^8$ [p/s/cm^2/sr]/[µW/cm^2]).

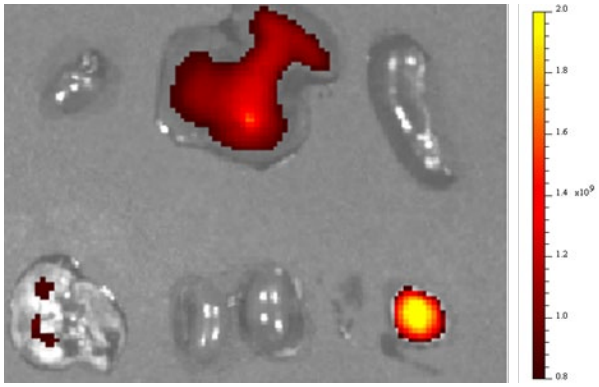

Figure S19. Representative *ex vivo* imaging of tumors and main organs in 4T1 tumor-bearing mice 24-hour post CuP-Ce6/Er intravenous injection. Color bar represents radiant efficiency([p/s/cm^2/sr]/[μW/cm^2]).

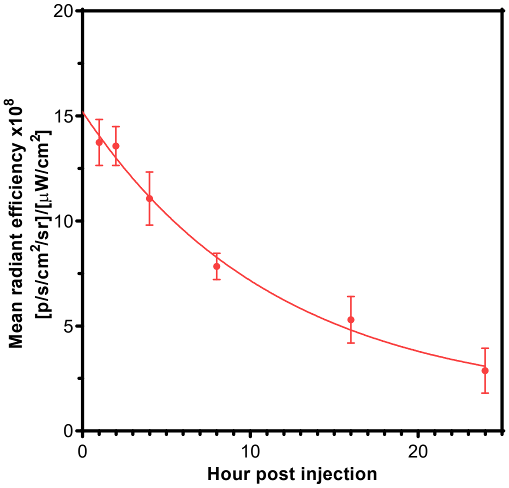

Figure S20. Time-dependent Ce6 fluorescence intensities in the plasma after CuP-Ce6/Er intravenous injection.

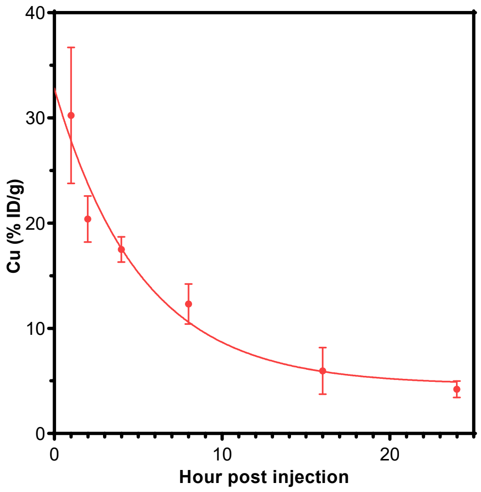

Figure S21. Time-dependent Cu concentrations in plasma after CuP-Ce6/Er intravenous injection.

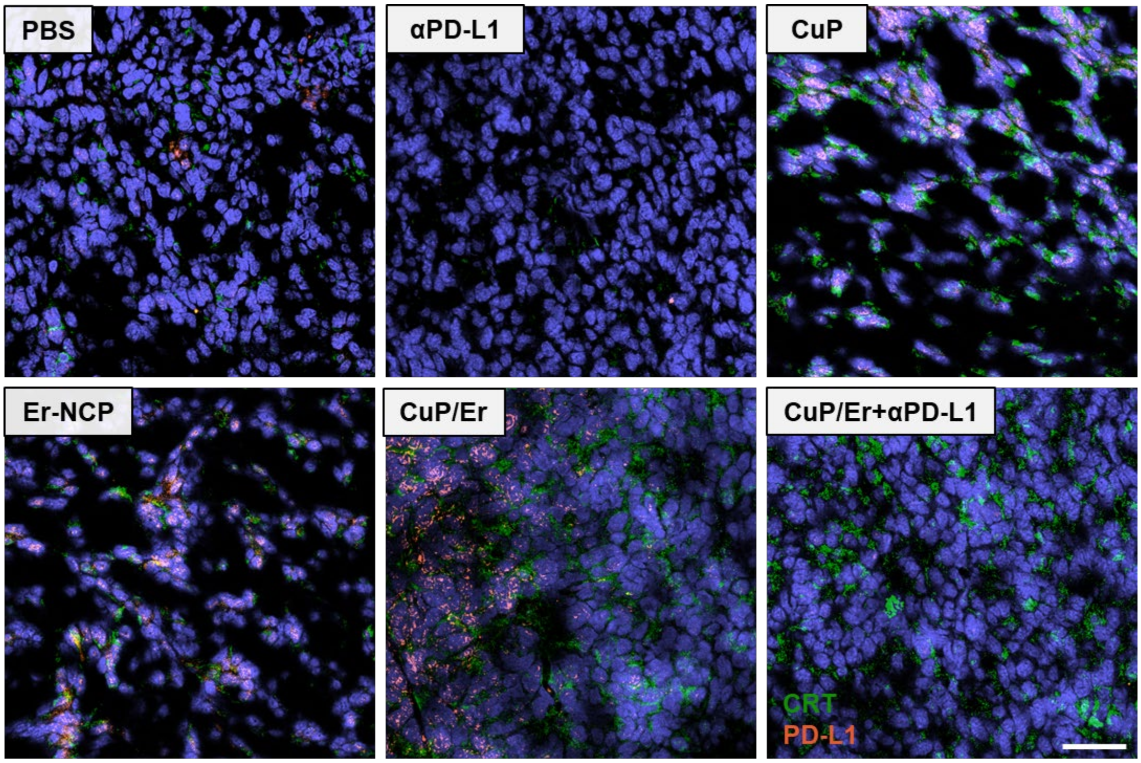

Figure S22. CRT and PD-L1 Immunofluorescence staining of 4T1 tumor cryo-sectioned slides after different treatments. Scale bar is 40 μm.

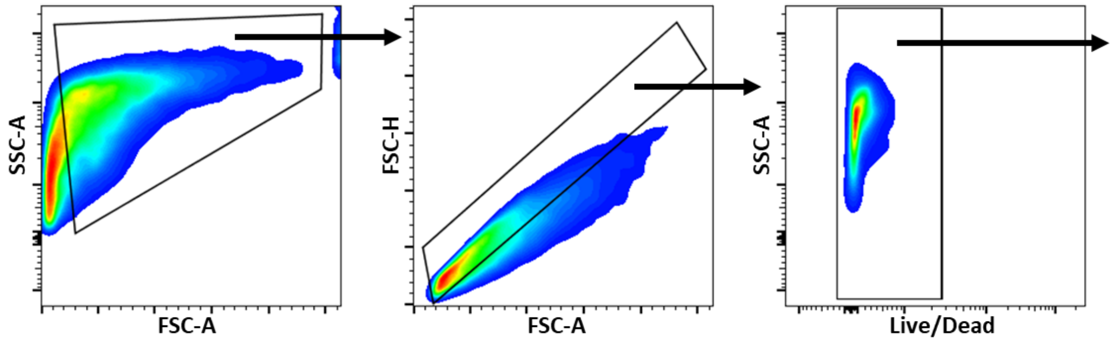

Figure S23. Gating strategies for single cells and live cells before further gating for immune cell populations.

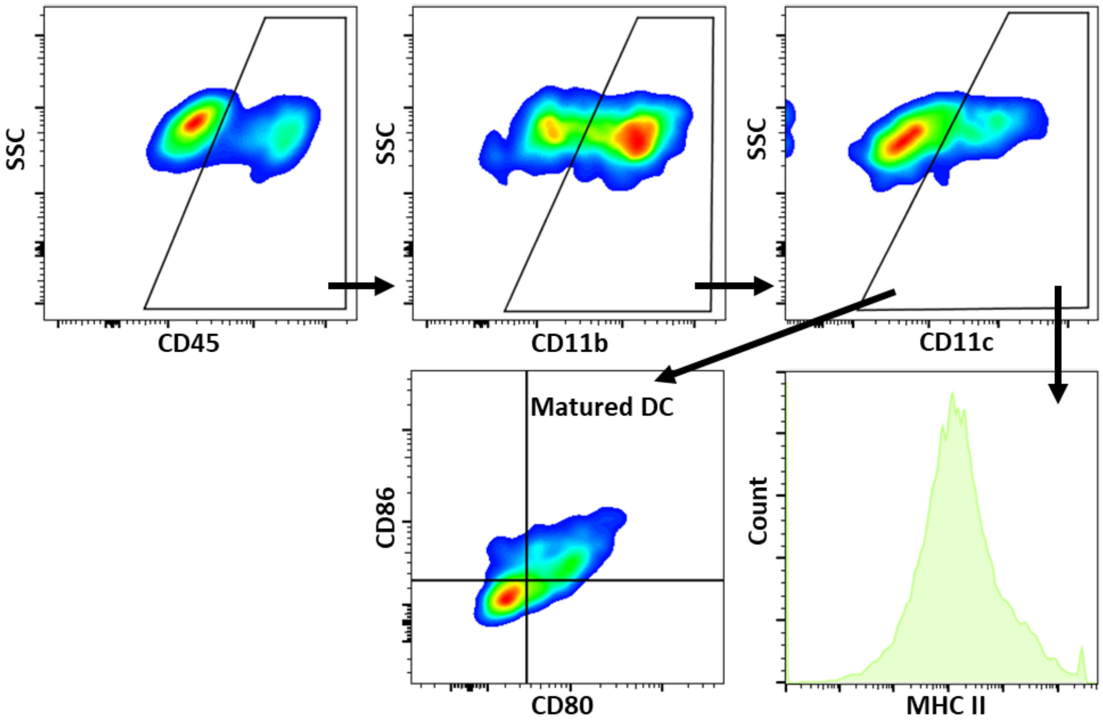

Figure S24. Gating strategies for matured DCs and relative intensity of MHC II in BMDCS co-incubated with tumor cells treated with different NCPs.

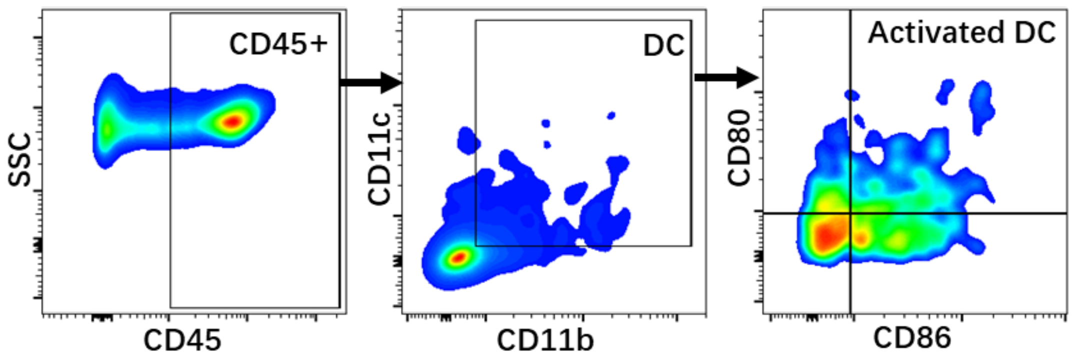

Figure S25. Gating strategies for DCs (CD45$^+$ CD11b$^+$ CD11c$^+$) and activated DCs (CD45$^+$ CD11b$^+$ CD11c$^+$ CD80$^+$CD86$^+$) in TDLNs.

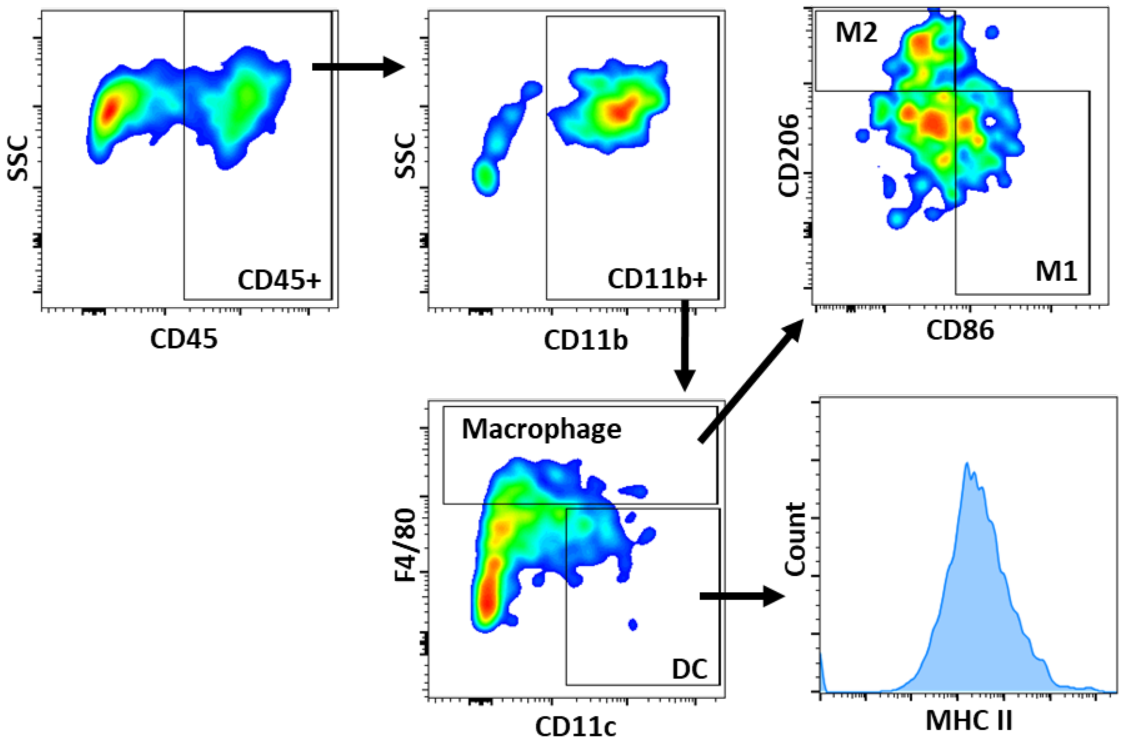

Figure S26. Gating strategies for DCs (CD45$^+$ CD11b$^+$ CD11c$^+$), relative intensity of MHC II of DCs, M1 (CD45$^+$CD11b$^+$F4/80$^+$CD86$^+$CD206$^-$) and M2 (CD45$^+$ CD11b$^+$F4/80$^+$CD206$^+$CD86$^-$) macrophages in tumors.

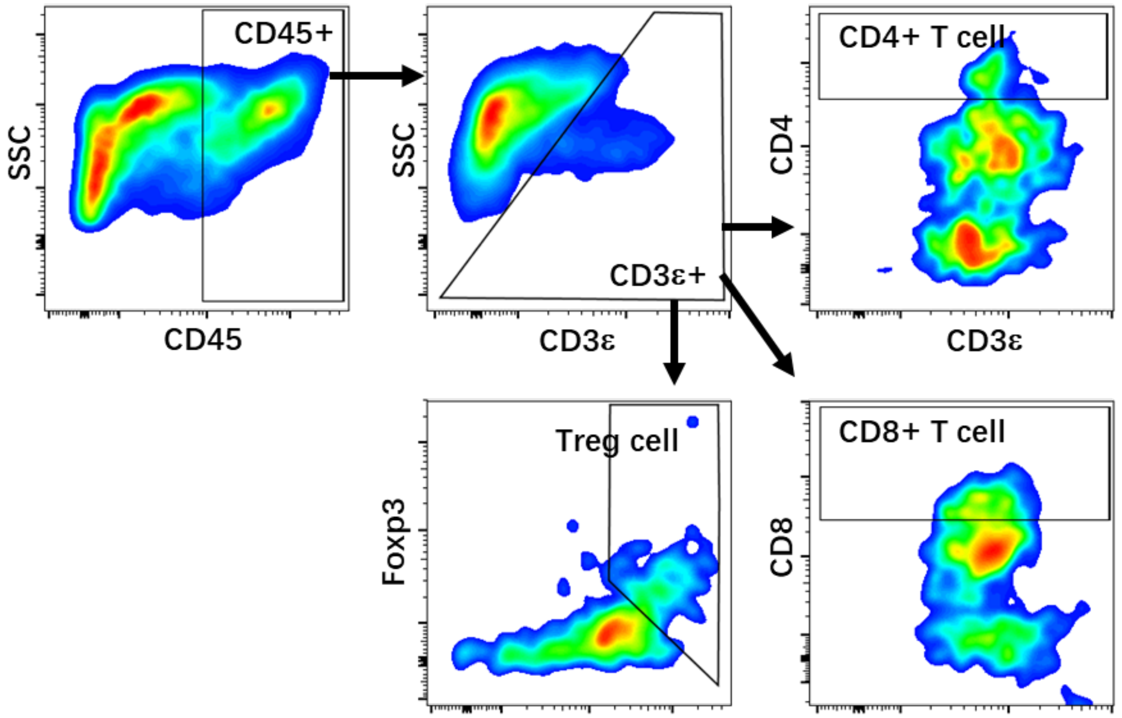

Figure S27. Gating strategies for cytotoxic T cells ($CD45^+CD3\varepsilon^+CD8^+$), helper T cells ($CD45^+CD3\varepsilon^+CD4^+$) and Treg cells ($CD45^+CD3\varepsilon^+CD4^+Foxp3^+$) in tumors.

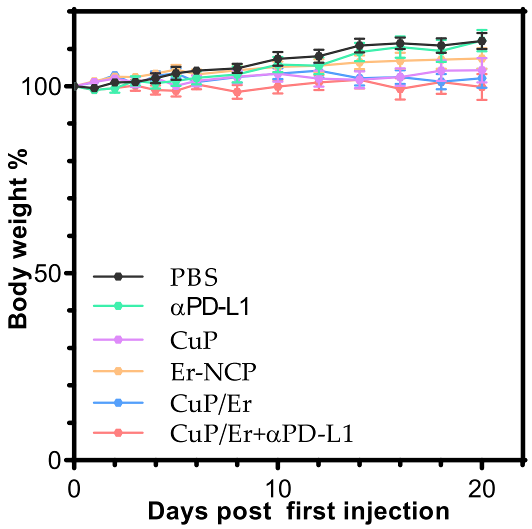

Figure S28. Relative wights of MC38 tumor bearing mice with different treatments.

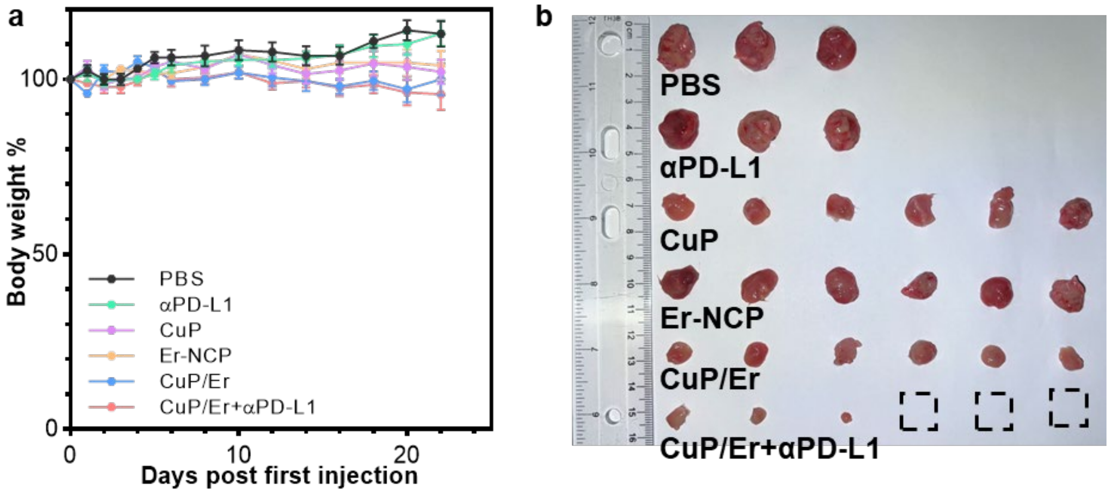

Figure S29. (a) Relative wights of 4T1 tumor bearing mice with different treatments. (b) Image of sectioned 4T1 tumors with different treatments at the endpoint. Data are represented as mean ± SD, n = 3 in PBS and α-PD-L1 groups and n=6 in other groups.

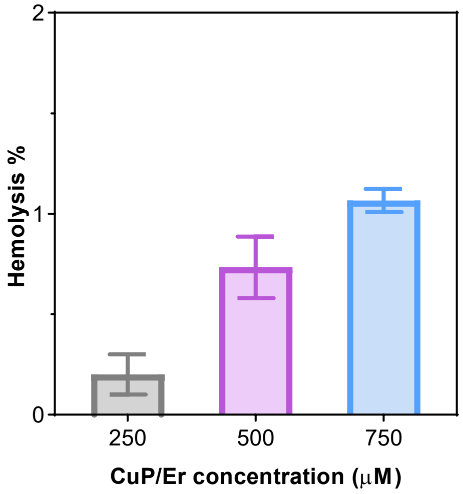

Figure S30. Hemolysis test results of CuP/Er treatment at different dose levels.

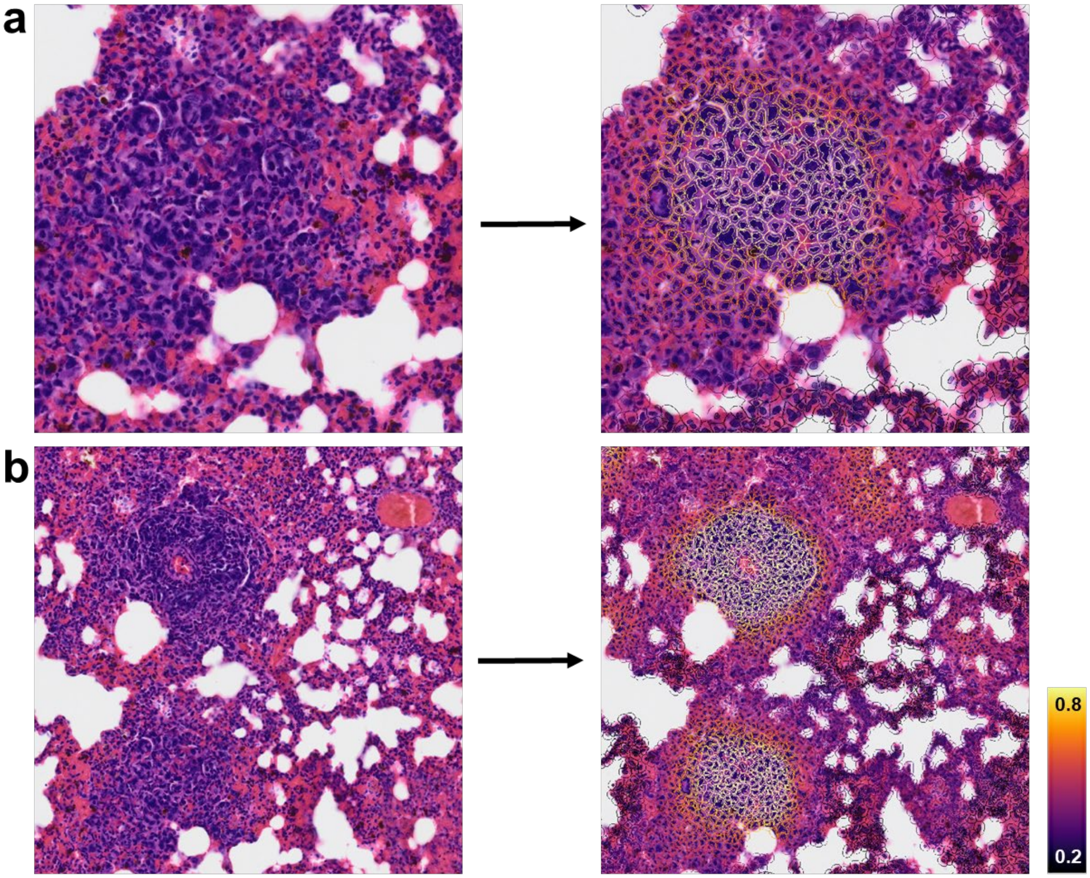

Figure S31. Representative figures of (a) cell detection of the nucleus and cell borders and (b) nucleus/cell ratio representing by colormap by QuPath software.

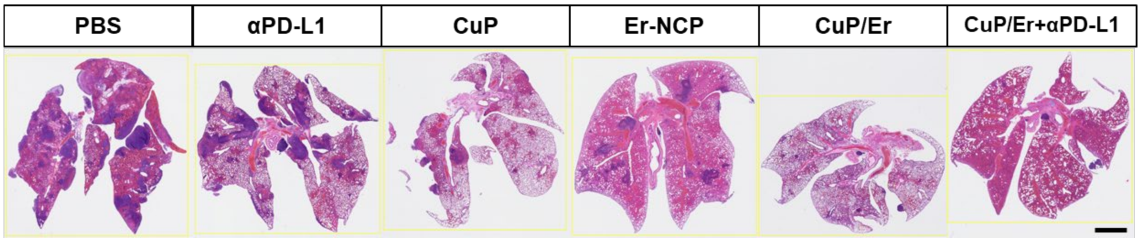

Figure S32. Representative H&E staining of lungs in 4T1 tumor bearing mice after different treatments. Scale bar is 2 mm.

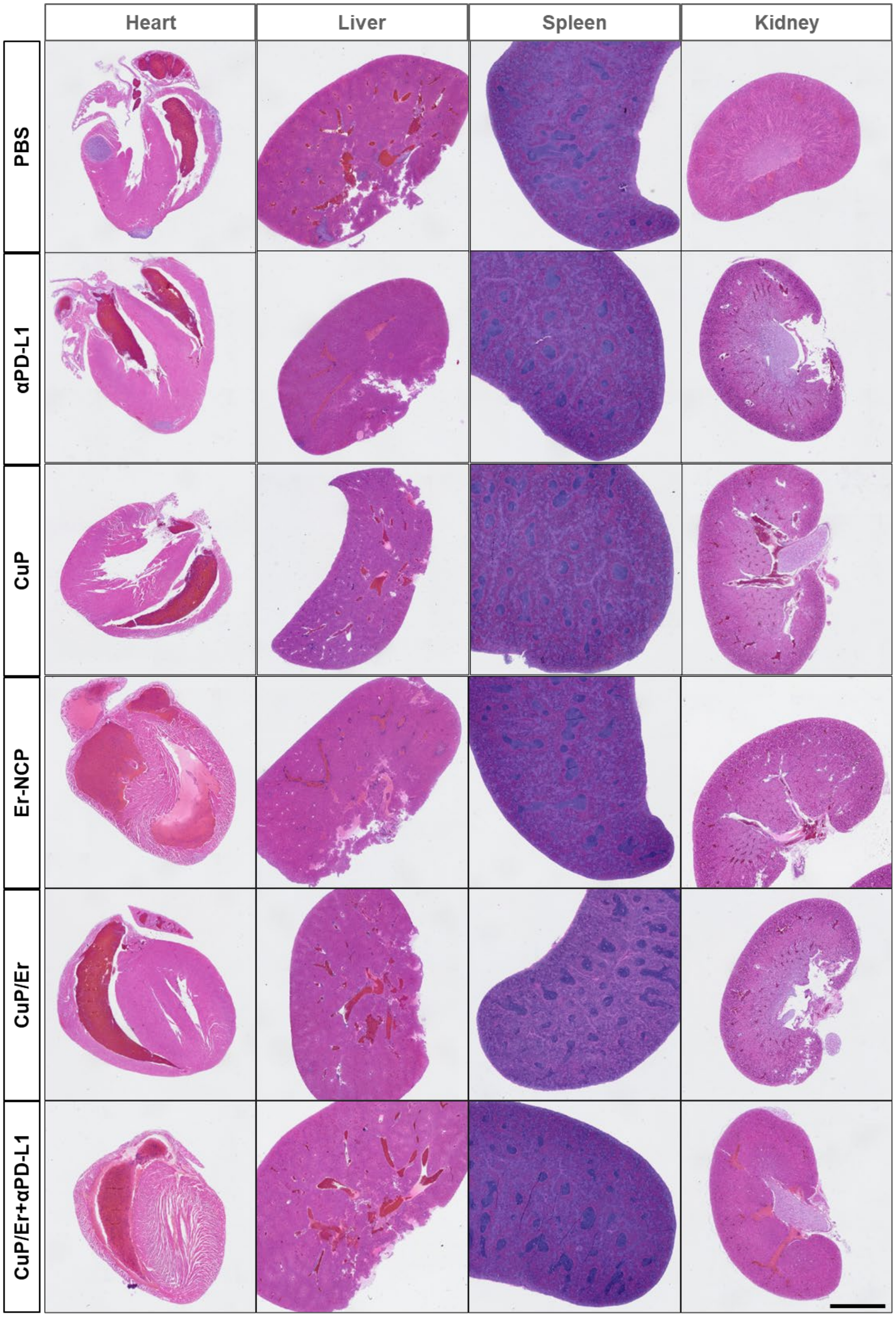

Figure S33. Representative H&E staining of major organs in 4T1 tumor bearing mice after different treatments. Scale bar is 2 mm.

3. Supporting Tables

Table S1. Representative GC results of O_2 after adding excess $KMnO_4$ solution to NCP CuP.

RetTime (min)	Gas Name	Area	Amount (ul)
0.670	H_2	-	-
0.845	O_2	1.486	1.947
2.237	CH_4	1.730e-1	2.156e-2
5.417	CO_2	1.248e-1	6.044e-2

Table S2. Ce6 pharmacokinetics of BALB/c mice following a single intravenous injection of CuP-Ce6/Er at a dose of 1mg/kg Ce6, 3.5 mg/kg Cu, 30 mg/kg erastin with one compartment model fitting. Data are represented as mean ± SD, n = 3.

Parameters	Unit	CuP-Ce6/Er
C_0	$\times 10^8$[p/s/cm^2/sr]/[μW/cm^2]	15.21 ± 1.32
C_{min}	$\times 10^8$[p/s/cm^2/sr]/[μW/cm^2]	1.37 ± 0.31
$t_{1/2}$	hour	7.96 ± 2.53
k	hour^{-1}	0.087 ± 0.021
$AUC_{0\text{-}inf\_obs}$	$\times 10^8$[p/s/cm^2/sr]/[μW/cm^2] ×hour	170.44 ± 1.39

Table S3. Cu pharmacokinetics of BALB/c mice following a single intravenous injection of CuP-Ce6/Er at a dose of 1mg/kg Ce6, 3.5 mg/kg Cu, 30 mg/kg erastin with one compartment model fitting. Data are represented as mean ± SD, n = 3.

Parameters	Unit	CuP-Ce6/Er
C_0	μg/ml	19.43 ± 3.55
C_{min}	μg/ml	2.78 ± 1.38
$t_{1/2}$	hour	3.57 ± 0.58
k	hour^{-1}	0.19 ± 0.03
$AUC_{0\text{-}inf\_obs}$	μg/ml×hour	158.98 ± 2.83

References

[1] X. Duan, C. Chan, N. Guo, W. Han, R. R. Weichselbaum, W. Lin, *J. Am. Chem. Soc.* **2016**, *138*, 16686-16695.

[2] D. Guanghua, A. D. Guido, F. Werner, G. Christoph, H. Volker, K. Reiner, K. Alexandra, T. Laura, A. F. Anna, D. Günther, *Radiat. Res.* **2011**, *176*, 706-715.

[3] P. Tsvetkov, S. Coy, B. Petrova, M. Dreishpoon, A. Verma, M. Abdusamad, J. Rossen, L. Joesch-Cohen, R. Humeidi, R. D. Spangler, J. K. Eaton, E. Frenkel, M. Kocak, S. M. Corsello, S. Lutsenko, N. Kanarek, S. Santagata, T. R. Golub, *Science.* **2022**, *375*, 1254-1261.

[4] B. Schmid, J. Schindelin, A. Cardona, M. Longair, M. Heisenberg, *BMC Bioinf.* **2010**, *11*, 274.
